# Real-World Driver Stress Recognition and Diagnosis Based on Multimodal Deep Learning and Fuzzy EDAS Approaches

**DOI:** 10.3390/diagnostics13111897

**Published:** 2023-05-29

**Authors:** Muhammad Amin, Khalil Ullah, Muhammad Asif, Habib Shah, Arshad Mehmood, Muhammad Attique Khan

**Affiliations:** 1 Department of Electronics, University of Peshawar, Peshawar 25120, Pakistan; 2 Department of Computer Science, Iqra National University, Peshawar 25000, Pakistan; 3 Department of Software Engineering, University of Malakand, Dir Lower, Chakdara 23050, Pakistan; 4 Department of Computer Science, King Khalid University, Abha 61421, Saudi Arabia; 5 Department of Mechanical Engineering, University of Engineering & Technology, Peshawar 25120, Pakistan; 6 Department of Computer Science, HITEC University, Taxila 47080, Pakistan

**Keywords:** driver stress recognition, multimodal data, deep learning, CNN, LSTM, fuzzy EDAS

## Abstract

Mental stress is known as a prime factor in road crashes. The devastation of these crashes often results in damage to humans, vehicles, and infrastructure. Likewise, persistent mental stress could lead to the development of mental, cardiovascular, and abdominal disorders. Preceding research in this domain mostly focuses on feature engineering and conventional machine learning approaches. These approaches recognize different levels of stress based on handcrafted features extracted from various modalities including physiological, physical, and contextual data. Acquiring good quality features from these modalities using feature engineering is often a difficult job. Recent developments in the form of deep learning (DL) algorithms have relieved feature engineering by automatically extracting and learning resilient features. This paper proposes different CNN and CNN-LSTSM-based fusion models using physiological signals (SRAD dataset) and multimodal data (AffectiveROAD dataset) for the driver’s two and three stress levels. The fuzzy EDAS (evaluation based on distance from average solution) approach is used to evaluate the performance of the proposed models based on different classification metrics (accuracy, recall, precision, F-score, and specificity). Fuzzy EDAS performance estimation shows that the proposed CNN and hybrid CNN-LSTM models achieved the first ranks based on the fusion of BH, E4-Left (E4-L), and E4-Right (E4-R). Results showed the significance of multimodal data for designing an accurate and trustworthy stress recognition diagnosing model for real-world driving conditions. The proposed model can also be used for the diagnosis of the stress level of a subject during other daily life activities.

## 1. Introduction

Successful driving activities always require both mental and physical skills [1,2,3]. Acute stress reduces the driver’s ability to fix hazardous situations, which causes significant damage to humans and vehicles every year [4,5,6,7,8]. Dangerous driving situations are triggered due to human errors, individual factors, and ambiance conditions [9]. According to the National Motor Vehicle Crash Causation Survey (NMVCCS) in the United States (US), human errors caused 94% of crashes alone, while vehicle defects, ambiance conditions, and other factors collectively caused 6% of crashes during 2005–2007 [10]. Human errors are linked to the driver’s perceptual conditions, so a complete understanding of these conditions is crucial for preventing traffic accidents. 

To detect and diagnose drivers’ different stress levels, physiological, physical, and contextual information are widely utilized [11]. Moreover, different traditional machine learning models based on handcrafted feature extraction methods are utilized for the classification of stress. Extracting the best features using these approaches is always a challenging task, as the quality of extracted features has a significant effect on the classification performance [12]. These approaches are laborious, ad hoc, less robust to noise, and need thorough skill [13]. To come through these challenges, deep learning models have been utilized to automatically produce complex nonlinear features reliably [14,15,16]. In addition to automatic feature extraction from raw data, these models offer noise robustness and better classification accuracy [17,18,19]. Different deep learning algorithms are used in recent research, e.g., CNN, RNN, DNN, and LSTM.

The models proposed in the current work are based on 1D CNN and hybrid 1D CNN-LSTM networks. The proposed models are separately trained using multiple physiological signals (SRAD) and multimodal data (AffectiveROAD) including physiological signals and other information about the vehicle, driver, and ambiance. Multimodal fusion of data based on deep learning approaches can be used to develop a precise driver stress level recognition model with improved performance and reliability.

Contributions of this research study include: (1) proposing 1D CNN and hybrid 1D CNN-LSTM-based real-world driver stress level recognition models using fused physiological signals (SRAD dataset) and fused multimodal data (AffectiveROAD dataset) and (2) ranking the assessment of the proposed models for the two and three levels of stress based on the fuzzy EDAS approach.

The organization of this research article is given below. Analysis of the existing stress recognition models is presented in Section 2. The proposed methodology is elaborated in Section 3 in terms of datasets, data pre-processing, architectures of the proposed CNN and hybrid CNN-LSTM models, and the fuzzy EDAS approach. Performance evaluation of the proposed models is conducted in Section 4. A fuzzy EDAS-based rank estimation of the proposed models for the driver’s two and three levels of stress is also presented in this section. Section 5 gives a detailed assessment of the proposed and existing stress recognition schemes. Finally, Section 6 concludes the paper and gives future directions to further explore this research area.

## 2. Related Work

This section provides a review of the existing work in the driver’s stress analysis domain and underscores the current contribution. Several driver stress level recognition schemes exist in the literature based on simulated and real-world driving environments. These schemes can be broadly categorized as conventional machine learning or deep learning models.

Several machine learning approaches have been proposed for real-world driver mental stress recognition based on different physiological signals. Dalmeida and Masala [20], Vargas-Lopez et al. [21], Khowaja et al. [8], Lopez-Martinez et al. [22], Haouij et al. [23], Chen et al. [4], Ghaderi et al. [24], Zhang et al. [25], and Healey and Picard [26] propose conventional machine models based on physiological signals obtained from the PhysioNet SRAD public database [27]. Unlike the previous studies, Rigas et al. [28] presented a real-world binary stress recognition model based on multimodal data, including physical and contextual data, in addition to physiological signals. On the other hand, Zontone et al. [29], Bianco et al. [30], Lee et al. [31], Lanatà et al. [32], and Gao et al. [33] proposed conventional machine learning models for driver stress recognition based on simulated driving situations. Lanatà et al. [32] and Lee et al. [31] presented driver stress recognition models based on multimodal data. Contrary to previous studies, Šalkevicius et al. [34], Rodríguez-Arce et al. [35], Can et al. [36], Al abdi et al. [37], Betti et al. [38], Siramprakas et al. [39], de Vries et al. [40], and Sun et al. [41] proposed stress recognition models during controlled, lab, semi-lab, and physical (such as sitting, standing, and walking) environments. Recent development in deep learning and machine learning models have shown good results in various applied domains that can be applied in driver stress detection [42,43].

All the mentioned studies are based on feature engineering techniques, and various conventional machine learning algorithms were employed to classify levels of stress. However, handcrafted features are less robust to noise and subjective changes, and need a considerable amount of time and hard work [8,13,19,34,35,44]. Moreover, capturing the features’ sequential nature is difficult due to the absence of explicit features and high dimensionality despite using complex feature selection methods. Likewise, the dependence of the model on past observations would make it impractical to process all the information due to the growing complexity. The feature-level multimodal fusion models proposed by Chen et al. [4], Healey and Picard [26], Haouij et al. [23], Lee et al. [31], Bianco et al. [30], Sun et al. [41], and Can et al. [36] mainly concentrate on pattern learning in individual signals instead of multiple simultaneous signals [18]. Thus, these models are inappropriate to obtain the nonlinear correlation across multiple signals appearing simultaneously. Various linear and non-linear methods employed in these conventional machine learning models have not been able to perform the vigorous investigation of such manifold time series signals [19].

To address the issues faced by conventional machine learning models, deep learning methods have been introduced. Deep learning models are developed based on signal preprocessing (noise filtering), designing a particular deep neural network based on the area of interest, network training, and model testing. Deep learning models learn and classify raw data using multilayer deep neural networks [45]. The last fully connected (FC) layers are utilized to obtain the final output. Contrary to feature engineering techniques used in conventional machine learning approaches, deep learning models automatically produce steady features [14,15]. Moreover, deep learning models are more robust to noise and achieve improved classification accuracy [19]. Different deep learning algorithms are used in recent research, e.g., the recurrent neural network (RNN), deep aeural network (DNN), LSTM, and CNN. Rastgoo et al. [11], Zhang et al. [46], Kanjo et al. [17], Lim and Yang [47], Yan et al. [48], Hajinoroozi et al. [49], and Lee et al. [50] presented different deep learning models to identify different driver states. Rastgoo et al. [11], Kanjo et al. [17], Lim and Yang [47], and Yan et al. [48] proposed deep learning models based on multimodal data. On the other hand, the models proposed by Hajinoroozi et al. [49] and Lee et al. [50] are based on physiological signals only. The stress recognition model proposed by Zhang et al. [46] is based on facial images only. Apart from driving scenarios, Masood and Alghamdi [51], Cho et al. [52], Seo et al. [53], Hwang et al. [54], and He et al. [55] proposed stress recognition models based on deep learning techniques and physiological signals in academic, workplace, and lab settings. Most of these studies including [46,49,50,52,53,54,55,56] are based on two levels of stress only. Moreover, the schemes presented by [46,50,52,55,56] are based on images. Likewise, the schemes proposed by [49,52,53,54,55,56] are either based on physiological signals or a single modality. On the other hand, the model proposed by [11] is based on multimodal data collected during simulated driving. 

The models proposed in this study are based on the fusion of multimodal data collected during real-world driving (SRAD and AffectiveROAD datasets). Moreover, these models are based on 1D CNN and 1D CNN-LSTM networks to detect driver’s two (stressed and relaxed) and three levels (low, medium, and high). The fuzzy EDAS approach is also used to find the performance ranks of the proposed models based on different classification metrics. 

## 3. Materials and Methods

The proposed unimodal and fusion models for real-world driver stress level recognition are based on physiological signals and deep learning approaches, such as CNN and hybrid CNN-LSTM. The proposed models are implemented in the latest MATLAB 2022a platform. The proposed stress recognition models are based on the fusion of ECG, HR, HGSR, FGSR, EMG, and RESP signals collected from the PhysioNet SRAD database, and breathing rate (BR), GSR, BVP, HR, TEMP, ACCEL, posture, and activity data are collected from AffectiveROAD database. Data input mechanisms used in this research are based on raw signals. These raw signals are preprocessed to obtain cleaned signals.

### 3.1. SRAD Dataset

The ECG, HR, GSR, EMG, and RESP signals analyzed in the current work belong to the SRAD PhysioNet public database [57]. Experiments were performed while driving a customized Volvo S70 series station wagon. Five different sensors were used to acquire physiological signals from the nine drivers during twenty-four drives. The sensors were connected to an embedded computer through an analog-to-digital converter (ADC). The ECG sensor was placed using a modified lead II configuration to decrease the motion artifacts. The EMG sensor was positioned on the shoulder near the trapezius muscle to record the emotional stress. Two GSR sensors were located on the driver’s sole and palm of the left foot and hand. Expansion of the chest cavity was used to measure the RESP signals through an elastic Hall effect sensor fastened around the diaphragm. 

All drives comprise rest, highway, and city driving phases on a specific route 31 km in length in Boston, US. These rest, highway, and city driving phases are assumed to trigger low, medium, and high levels of stress, respectively. Initially, the drivers are informed about the travel plan and compliance with certain guidelines regarding the speed limits and tuning out the radio. To avoid rush hours, all drives were performed in the midmorning and afternoon. Two rest intervals of 15 min in the parking area were added at the start and end of each drive to collect the driver’s low-stress baseline. Due to stop-and-go traffic in the city area, drivers usually observe high-stress situations. After passing the toll booth, the city road then turns into the highway. Uninterrupted highway driving normally indicates medium-stress conditions. The trip completes after returning to the starting position using the same highway and city routes. The total length of all drives varies from 50 to 90 min, including two 15 min rest intervals.

**Figure 1:**
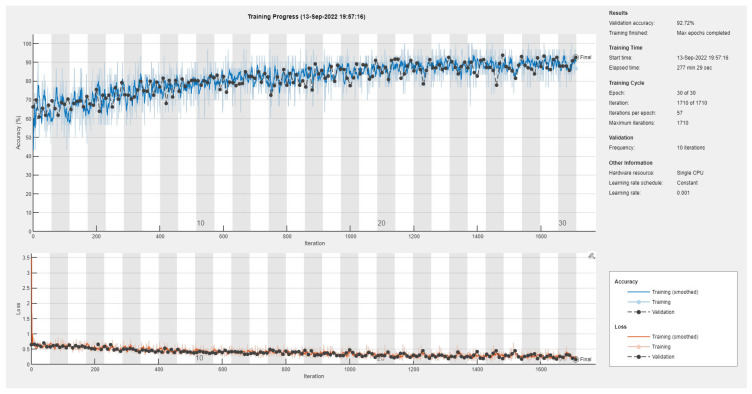
Training graph of the CNN model for the two-stress class based on the SRAD dataset.

**Figure 2:**
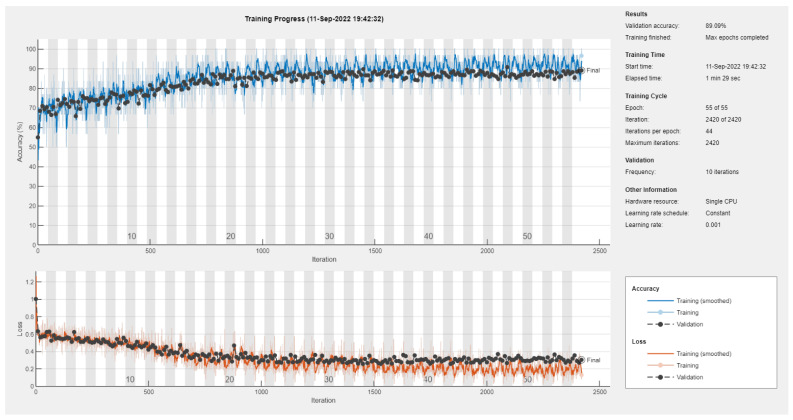
Training graph of the CNN model for the two-stress class based on the BH dataset.

**Figure 3:**
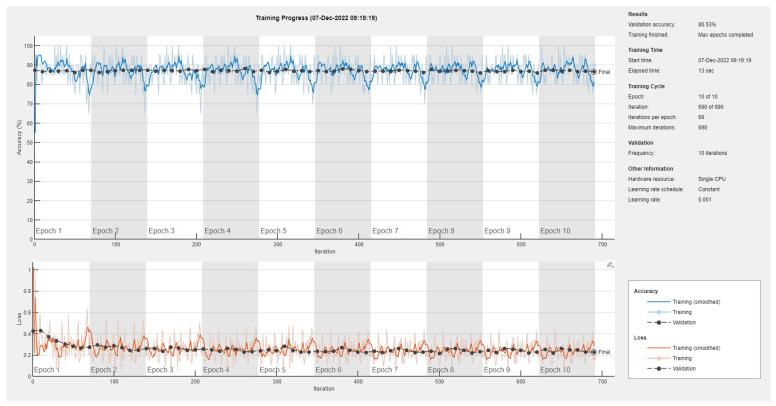
Training graph of the CNN model for the two-stress class based on the E4-L dataset.

**Figure 4:**
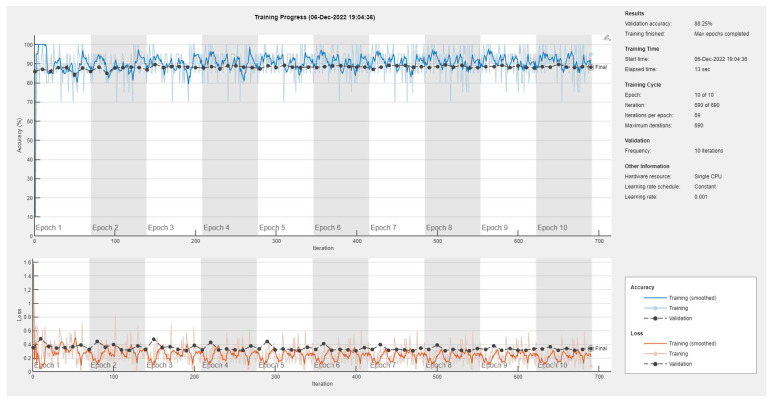
Training graph of the CNN model for the two-stress class based on the E4-R dataset.

**Figure 5:**
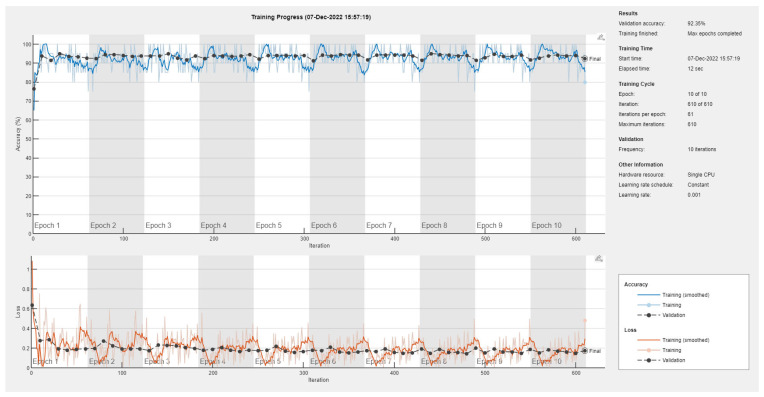
Training graph of the CNN model for the two-stress class based on the E4-(L+R) datasets.

The dataset contains information about 17 drives, but some drives have incomplete signals and markers. These incomplete drives are removed from the experiments. Figure 1, Figure 2, Figure 3, Figure 4 and Figure 5 separately show the ECG, HR, GSR, EMG, and RESP waveforms for the three levels of stress. The figures show that all five signals have distinct waveforms for the three levels of stress.

### 3.2. AffectiveROAD Database

Experiments were performed using wireless sensors networked together inside different cars to collect physiological signals and additional information about the vehicle, driver, and ambiance. The Zephyr Bio-harness (BH) chest strap was placed on the driver’s chest to collect HR, breathing rate (BR), posture, and activity information. Two Empatica E4-Left (E4-L) and E4-Right (E4-R) wearable devices were mounted on the driver’s left and right arms to capture GSR, BVP, inter-beat interval (IBI), HR, TEMP, and ACCEL data. The Intel Edison developer kit-based environmental platform was placed in the car’s rear seat for collecting luminosity, temperature, pressure, and humidity information. A sound meter and microphone were used to obtain sound amplitude and audio signal. Two cameras were placed on the windshield of the car to record inside and outside events. A real-time continuous subjective metric was prepared by an experimenter during each drive to monitor the driver’s stress level. The stress metric along with two video recordings were then used by the drivers to correct and validate the experimenter’s ratings.

All drives comprise rest, highway, and city driving phases on a fixed route 31 km in length in the Grand Tunis area. Fourteen driving experiments were performed by 10 experienced drivers with valid driver’s licenses. Each drive included two 15 min rest periods at the start and end of the session. The whole experiment normally took about 86 min to travel through the zone, city1, highway, and city2, and then travel back in the opposite direction to reach the starting point. The rest, highway, and city drives were supposed to yield low, medium, and high levels of stress, respectively.

### 3.3. Pre-Processing

Physiological data are normally derived from the human body in the form of low-amplitude signals with different frequency ranges. These signals are mostly polluted by different noises and artifacts. To model the driver’s stress levels accurately, it is necessary to preprocess the ECG, HR, HGSR, FGSR, EMG, and RESP signals first. 

ECG signals normally contain different unwanted components including baseline wander, powerline interference (PLI), and high-frequency EMG noise [58]. Moreover, the PLI adds 50–60 Hz noise components in ECG signals [59]. Likewise, high-frequency EMG noise components caused by muscle contractions contaminate the ECG signals [58]. HR signals are commonly derived from ECG signals, so they inherit some noise and artifacts from ECG signals. To remove the baseline wander and other artifacts form ECG signals, a band-pass Butterworth filter (5–15 Hz) was used to eliminate the baseline wander. Similarly, a finite impulse response (FIR), Notch filter (59–61 Hz), and FIR band-pass filter (1.5–150 Hz) were used for noise removal. The min–max normalization approach is then utilized to remove the subject-specific baseline and motion artifacts.

A GSR signal is an effective stress measure that is comparatively less susceptible to noise [60]. Yet, the authors of [61] used a low-pass filter (4 Hz) and a Gaussian filter for denoising the GSR signal. These filters are used in this study too to obtain cleaned GSR signals. The signals are also normalized to the maximum value.

The EMG signal is contaminated by several unwanted signals including motion artifacts, PLI, capacitive effects, and ECG artifact signals. In this work, a band-pass Butterworth filter (0.5–500 Hz) is used to remove the low- and high-frequency noises in the EMG signals. Likewise, PLI is eliminated using a 60 Hz Notch filter. The min–max normalization is performed to remove the subject-specific baseline and motion artifacts. EMG signals in the SRAD dataset were initially collected at a lower sampling frequency of 495 Hz. Although, the EMG signal contains information up to 450 Hz. As per the Nyquist theorem, at least a 900 Hz sampling frequency is required for the EMG signals. 

The RESP signal is normally polluted by different undesirable signals including baseline wander, PLI, and motion artifacts. To remove high-frequency noise and baseline signal from the RESP signal, we applied Butterworth high-pass (0.05 Hz) and low-pass (0.70 Hz) filters, respectively.

### 3.4. 1D CNN Models

CNN-based models were originally developed to learn the internal representation of 2D images and then classify them into certain output classes. The same approach can be utilized for automatic feature learning and classification of time series sequenced data [62]. A 1D CNN uses several filters to perform 1D convolution (Conv1D) operations for constructing feature maps from such data. These networks can better match the 1D characteristic of different physiological signals. Increasing the convolutional layers can help CNN models to gradually extract unique and vigorous higher-level features. The 1D CNN models used in this research are based on the signal fusion of the SRAD and AffectiveROAD datasets for both two-stress and three-stress classes. Thus, all signals in the SRAD dataset are combinedly trained using the 1D CNN model. The AffectiveROAD dataset consists of multimodal data collected using BH, E4-L, and E4-R devices. So, different 1D CNN models are trained using the BH, E4-L, E4-R, E4-(Left+Right) (E4-(L+R)), and BH+E4-(L+R) datasets. A sliding window approach is used to convert each cleaned signal into equal size segments. These segments are then fed to a 1D CNN as new training data. The CNN-based driver stress recognition performs both feature learning and classification tasks. 

A 1D CNN architecture is defined using multiple Conv1D blocks each containing convolution, ReLU, and layer normalization (LN) layers. One-dimensional CNN architectures based on SRAD, E4-L, E4-R, E4-(L+R), BH, and BH+E4-(L+R) datasets are shown in Table 1. The convolution layer utilizes trainable filters (kernels) to convolve the low-level features of each segment or the previous layer’s output to produce a feature map. The number of filters in each convolutional block is set differently depending on the dataset. Causal padding is used in all convolutional layers to produce outputs with the same length. It pads the layer’s input with zeros to predict the values of early time steps in the frame. The convolutional layer is followed by the ReLU layer, which is based on a piecewise linear function. This function returns output for positive inputs and is zero otherwise, thus alleviating the vanishing gradient problem [63]. Moreover, the function adds nonlinearity to the model to learn complex patterns in the data. A GAP layer is added after the four convolutional blocks to produce a single vector output. This layer finds the average output of each feature map generated by the convolutional layers and provides a substitute for the flattening block. The last three layers including FC, softmax, and classification layers perform the classification task. The vector output of the GAP layer is fed to the FC layer, which is also known as the hidden layer. The FC layer is used to map the output classes to a vector of probabilities. The output of the FC layer is utilized by the softmax layer to perform the final classification decision by allocating probabilities to low, medium, and high classes of stress. The final classification layer uses a cross-entropy loss function to evaluate the performance of the classification model. An increase in cross-entropy loss reflects the divergence of the predicted probability from the actual label and vice versa. The classification layer assumes the number of classes from the FC and softmax layers.

#### Network’s Training

Before starting the training process, several parameters need to be settled. These pa-rameters include the training algorithm, mini-batch size, validation frequency, initial learning rate, and maximum epochs. Parameter settings for different 1D CNN models are shown in Table 1.

A training algorithm is used to reduce the loss function of a learning model iterative-ly based on a training dataset. Adaptive moment estimation (Adam) is used as a training algorithm. It combines the benefits of RMSProp and AdaGrad by calculating the individ-ual adaptive learning rates based on the parameters estimated for the first and second moments of gradients. The mini-batch represents a subset of segments used in a single training iteration. Min-batch size is set to a small value to ensure the uniform distribution and utilization of the full dataset during a single epoch. The validation frequency repre-sents the training iterations between evaluations of validation metrics, while training iter-ation is a single step performed by an optimization algorithm to reduce the loss function for a mini-batch. The network’s validation frequency is set to 10. The epoch represents the maximum iterations completed by the optimization algorithm to reduce the loss function for the entire dataset. All datasets are divided into 80% for training data and 20% for vali-dation data.

### 3.5. Hybrid 1D CNN-LSTM Models

The LSTM is a particular type of RNN developed by Hochreiter and Schmidhuber [64]. It is useful to discover and remember long sequences of data efficiently. Generally, the LSTM is a chain of repeating cells of neural networks, such as a RNN, but both have different cell structures. The RNN’s cell consists of a single neural network based on tangent hyperbolic function, while LSTM’s cell has four interacting neural network layers based on sigmoid functions and pointwise multiplication operations. The LSTM has several cells connected to each other horizontally. Information can be added or removed from the cell state using four different gates. Each LSTM cell consists of an input gate, cell state gate, forget gate, and output gate. The forget gate is based on the sigmoid function, which determines which information needs to be forgotten from the cell state. The information is removed if the gate generates zero output and it is retained if the gate produces one output. The cell state gate determines the cell state based on the new information. First, the input gate based on the sigmoid function determines the values to be updated. Next, a vector is created for the new candidate values by the tangent hyperbolic activation function. The cell state is updated by combining the results of the two functions. To generate the output of a cell, the output gate first applies the sigmoid function to the part of a cell state. Next, a tangent hyperbolic function is applied to the cell state and the resulting value is multiplied by the output of the sigmoid function.

The hybrid CNN-LSTM model utilizes both 1D CNN and LSTM networks to classify sequenced data. In such a model, the CNN is used as a front end to extract features from physiological data followed by the LSTM layers to perform learning and classification tasks. The hybrid CNN-LSTM model has a similar architecture to the CNN model with additional LSTM cells after the FC layers. The architecture of the CNN model is already discussed in the previous section. The hybrid 1D CNN-LSTM architectures based on the SRAD, BH, E4-L, E4-R, E4-(L+R), and BH+E4-(L+R) datasets are shown in Table 2. Moreover, parameter settings for the proposed models are also shown in the same table.

### 3.6. Fuzzy EDAS Approach

The fuzzy EDAS approach is used to evaluate the performance of the proposed real-world driver stress level detection models based on different modalities. This approach performs the rank estimation of the proposed models in terms of accuracy, recall, precision, F-score, and specificity. Fuzzy EDAS is an eight-step process where each step performs some sort of calculations, which in turn is used by the coming steps, as elaborated below: 

Step 1: First, the “solution of the average value (ψ)” is calculated for all matrices, as shown mathematically in the equation below:

(1) $$(\psi )=[\psi_{\beta }{]}_{1\times \delta }$$

where:

(2) $$\left(\psi_{\beta }\right)=\frac{\sum_{i=1}^{x}X_{\alpha \beta }}{x}$$

The aggregate solution of Equations (1) and (2) can be found as the average value (ψ) against every criterion’s estimated quantity for each performance metric.

Step 2: The positive distances from the average $\left(P_{I}\right)$ of each signal for the driver’s each stress level is calculated using the following equation:

(3) $$P_{I}=[{\left(P_{I}\right)}_{\alpha \beta }{]}_{\delta \times \delta }$$

The ${{(P}_{I})}_{\alpha \beta }$ in Equation (3) is the positive distance of β_th_ model from the average value for the α_th_ parameter. It can be found using either of two ways. If β_th_ criterion is more favorable, then it is calculated using the equation below:

(4) $${{(P}_{I})}_{\alpha \beta }=\frac{Maximum(0,(A_{V_{\beta }}-X_{\alpha \beta }))}{A_{V_{\beta }}}$$

On the other hand, if the β_th_ criterion is not favorable, it is calculated by the following equation:

(5) $${{(P}_{I})}_{\alpha \beta }=\frac{Maximum(0,(X_{\alpha \beta }-A_{V_{\beta }}))}{A_{V_{\beta }}}$$

Step 3: The negative distances from the average ${(N}_{I}$) of each signal for the driver’s stress level is calculated using the following equation:

(6) $${(N}_{I})=[{{(N}_{I})}_{\alpha \beta }{]}_{\delta \times \delta }$$

The ${{(N}_{I})}_{\alpha \beta }$ in Equation (6) is the negative distance of the β_th_ model from the average value for the α_th_ parameter. It can be found using either of two ways. If the β_th_ criterion is more favorable, then it is calculated using the equation below:

(7) $${{(N}_{I})}_{\alpha \beta }=\frac{Maximum(0,(A_{V_{\beta }}-X_{\alpha \beta }))}{A_{V_{\beta }}}$$

On the other hand, if the β_th_ criterion is not favorable, it is calculated by the following equation:

(8) $${{(N}_{I})}_{\alpha \beta }=\frac{Maximum(0,(X_{\alpha \beta }-A_{V_{\beta }}))}{A_{V_{\beta }}}$$

Step 4: The weighted sum of ${{(P}_{I})}_{\alpha \beta }$ is calculated using the following equation:

(9) $${{(SP}_{I})}_{\alpha }=\sum_{\beta =1}^{x}y_{\beta }{{(P}_{I})}_{\alpha \beta }$$

The aggregate ${(P}_{I})$ is estimated for each signal evaluated using the proposed model for each stress level.

Step 5: The weighted sum of ${{(N}_{I})}_{\alpha \beta }$ is calculated using the following equation:

(10) $${{(SN}_{I})}_{\alpha }=\sum_{\beta =1}^{x}y_{\beta }{{(N}_{I})}_{\alpha \beta }$$

The aggregate ${(N}_{I})$ is estimated for each signal evaluated using the proposed model for each stress level.

Step 6: The normalized values of ${{(SP}_{I})}_{\alpha }$ and ${{(SN}_{I})}_{\alpha }$ of each signal for the driver’s stress level are found using the following two equations:

(11) $$N{{(SP}_{I})}_{\alpha }=\frac{{{(SP}_{I})}_{\alpha }}{{maximum}_{\alpha }({{(SP}_{I})}_{\alpha })}$$

(12) $$N{{(SN}_{I})}_{\alpha }=1-\frac{{{(SN}_{I})}_{\alpha }}{{maximum}_{\alpha }\left({{(SN}_{I})}_{\alpha }\right)}$$

Step 7: The appraisal score (λ) of each signal for the driver’s stress level is calculated using the equation given below:

(13) $$\lambda_{\alpha }=\frac{1}{2}(N{{(SP}_{I})}_{\alpha }-N{{(SP}_{I})}_{\alpha })$$

The appraisal score $\left(\lambda_{\alpha }\right)$ lies are given as $0\leq \lambda_{\alpha }\leq 1$.

Step 8: Each signal for the driver’s stress level is ranked according to the decreasing values of the appraisal score $\left(\lambda_{\alpha }\right)$. Thus, the signal with the lowest appraisal score $\left(\lambda_{\alpha }\right)$ for a particular stress level has the highest performance among the other signals.

## 4. Results

The SRAD and AffectiveROAD datasets are randomly distributed into two groups, with 85% and 15% for training and validation, respectively. Results are acquired for the 1D CNN and 1D CNN-LSTM models trained using the SRAD, BH, E4-L, E4-R, and BH+E4-(L+R) datasets. A performance assessment of the proposed driver stress recognition models for the low, medium, and high classes of stress is carried out using different classification metrics. These performance metrics include accuracy (ACC), recall (RCL), precision (PRC), F-score (F1), and specificity (SPC).

### 4.1. Models’ Evaluation for the Two-Stress Class

**Figure 6:**
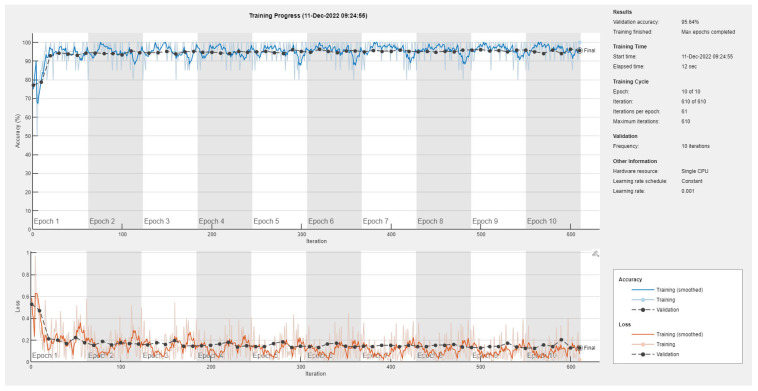
Training graph of the CNN model for the two-stress class based on the BH+E4-(L+R) datasets.

**Figure 7:**
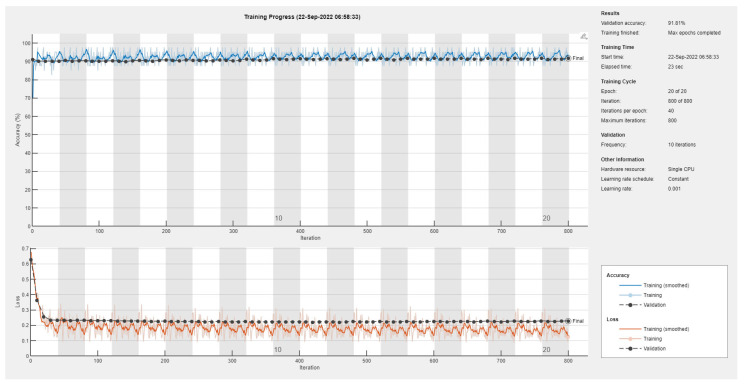
Training graph of the hybrid CNN-LSTM model for the two-stress class based on the SRAD dataset.

**Figure 8:**
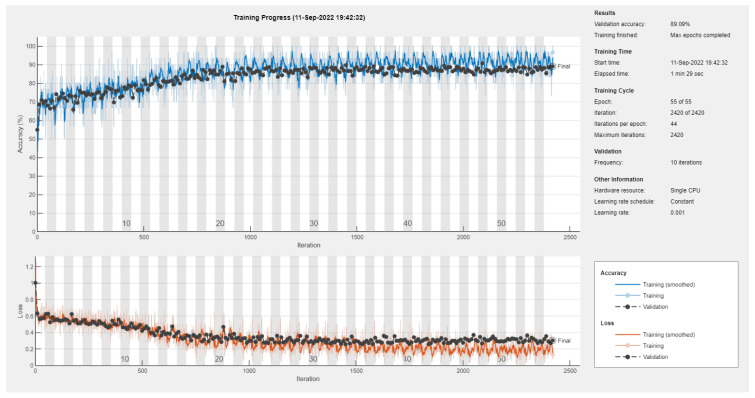
Training graph of the hybrid CNN-LSTM model for the two-stress class based on the BH dataset.

**Figure 9:**
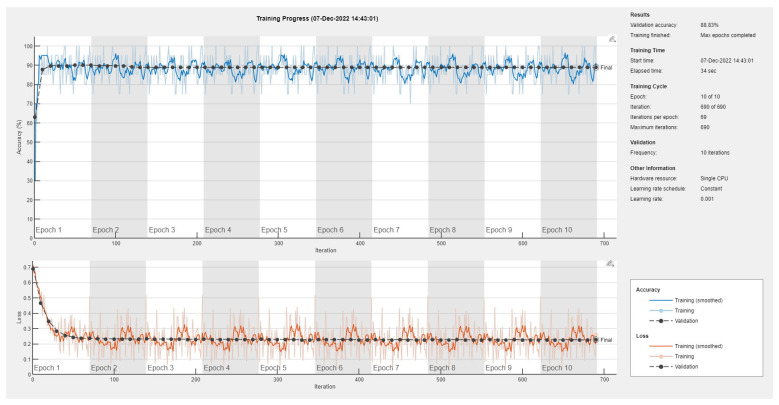
Training graph of the hybrid CNN-LSTM model for the two-stress class based on the E4-L dataset.

**Figure 10:**
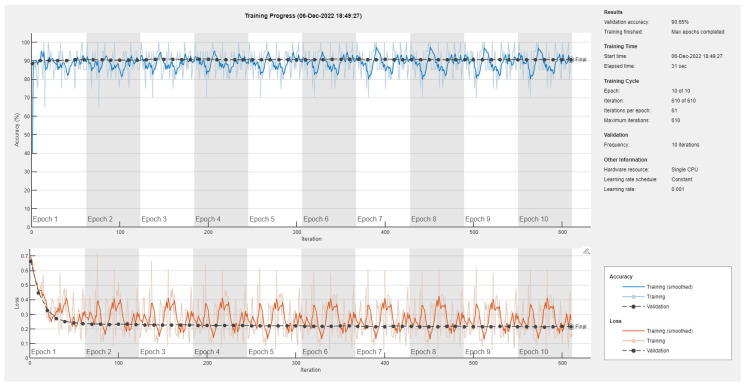
Training graph of the hybrid CNN-LSTM model for the two-stress class based on the E4-R dataset.

**Figure 11:**
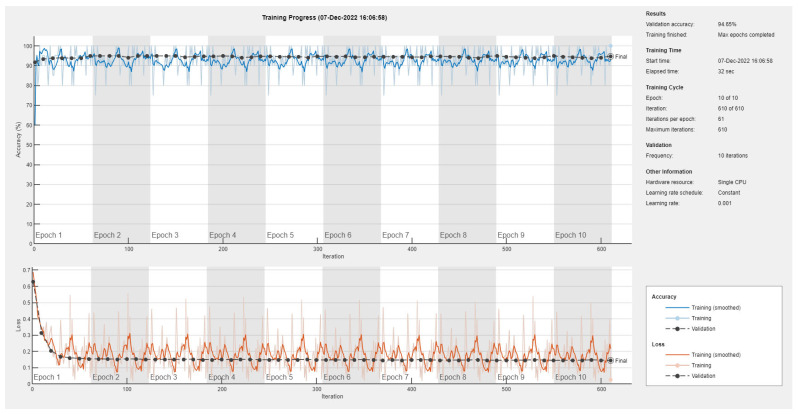
Training graph of the hybrid CNN-LSTM model for the two-stress class based on the E4-(L+R) datasets.

**Figure 12:**
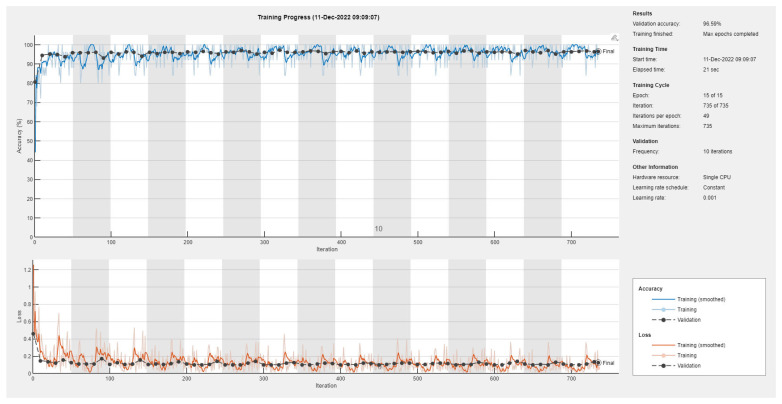
Training graph of the hybrid CNN-LSTM model for the two-stress class based on the BH+E4-(L+R) datasets.

Results of the proposed driver stress recognition models for the two-stress class are shown in Table 3. These results are based on the training data obtained from the SRAD and AffectiveROAD datasets for real-world driving. The training graphs of the proposed CNN models are shown in Figure 1, Figure 2, Figure 3, Figure 4, Figure 5 and Figure 6. Similarly, the training graphs of the proposed hybrid CNN-LSTM models are shown in Figure 7, Figure 8, Figure 9, Figure 10, Figure 11 and Figure 12. Results show that the BH+E4-(L+R)-based CNN model outperformed other models based on the SRAD, Bio BH, E4-L, E4-R, and E4-(L+R) datasets by 2.9%, 6.5%, 9.1%, 7.3%, and 3.25%, respectively, with an overall validation accuracy of 95.6% for the two-stress class. The proposed BH+E4-(L+R)-based hybrid CNN-LSTM model outperformed other models based on SRAD, BH, E4-L, E4-R, and E4-(L+R) datasets by 4.79%, 1.1%, 7.76%, 5.94%, and 1.94%, respectively, with an overall validation accuracy of 96.59% for the two-stress class.

**Figure 13:**
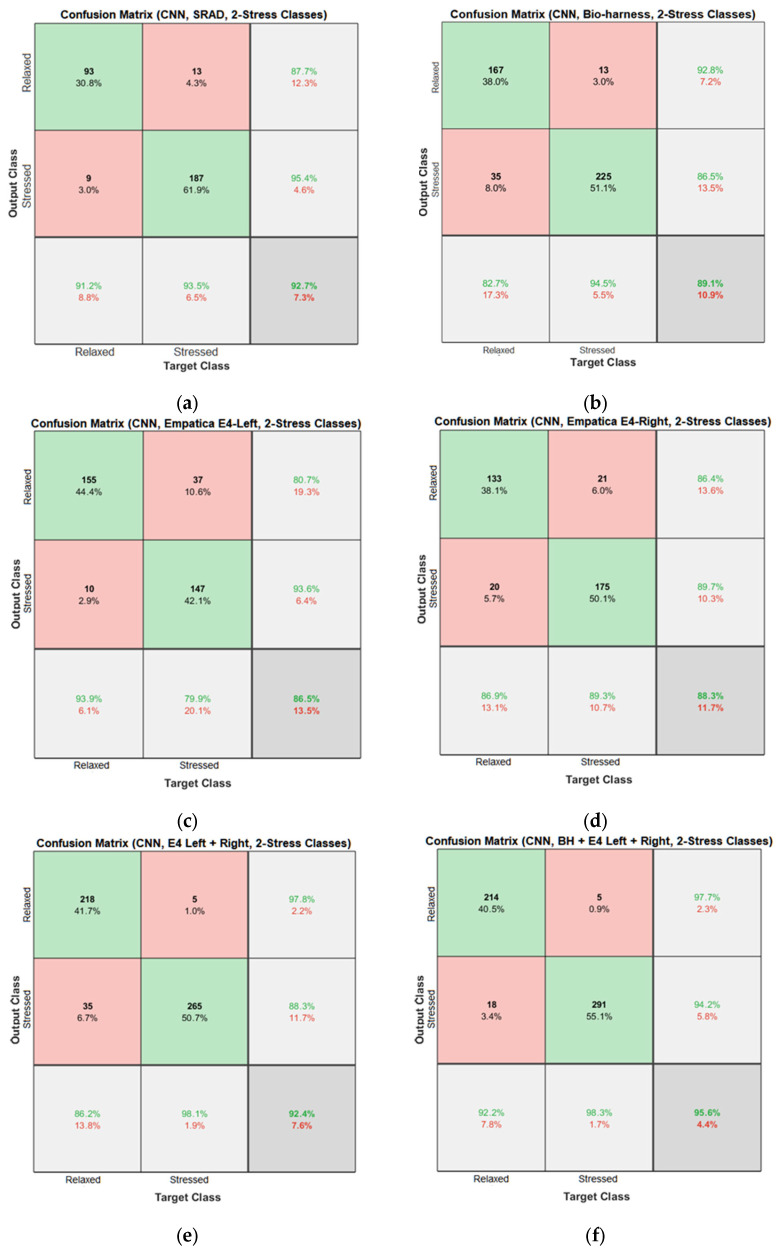
Confusion matrices of the CNN models for the two-stress class based on: (**a**) the SRAD dataset; (**b**) BH dataset; (**c**) E4-L dataset; (**d**) E4-R dataset; (**e**) E4-(L+R) datasets; (**f**) BH+E4-(L+R) datasets.

**Figure 14:**
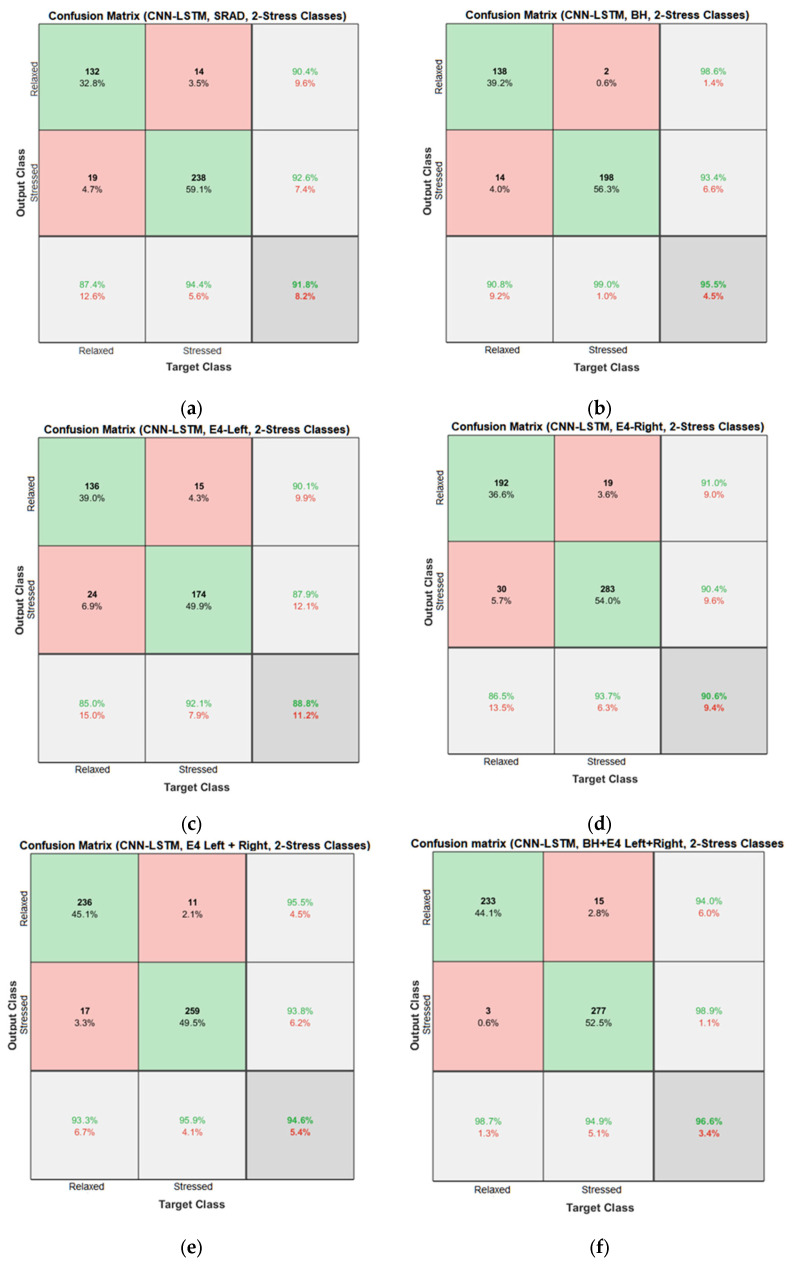
Confusion matrices of the hybrid CNN-LSTM models for the two-stress class based on: (**a**) the SRAD dataset; (**b**) BH dataset; (**c**) E4-L dataset; (**d**) E4-R dataset; (**e**) E4-(L+R) datasets; (**f**) BH+E4-(L+R) datasets.

Confusion matrices of the proposed CNN and hybrid CNN-LSTM models are shown in Figure 13 and Figure 14. In Figure 13f, 214 relaxed instances are predicted correctly, while 5 relaxed instances are incorrectly predicted as stressed by the model. Thus, the total correct prediction for the relaxed class is 97.7%. Similarly, for the stressed class, 291 out of 309 instances are correctly predicted, which amounts to a total accuracy of 94.2% for the stressed class. In Figure 14f, 233 relaxed instances are predicted correctly, while 15 relaxed instances are incorrectly predicted as stressed by the model. Thus, the total correct prediction for the relaxed class is 94%. Similarly, for the stressed class, 277 out of 280 instances are correctly predicted, which amounts to a total accuracy of 98.9% for the stressed class.

### 4.2. Models’ Evaluation for the Three-Stress Class

**Figure 15:**
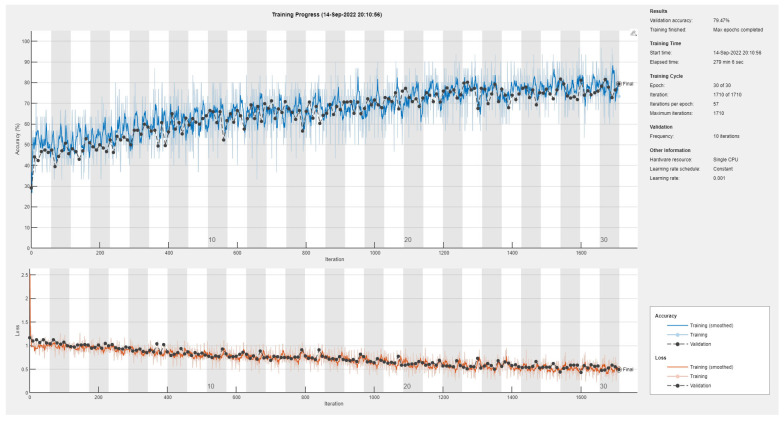
Training graph of the CNN model for the three-stress class based on the SRAD dataset.

**Figure 16:**
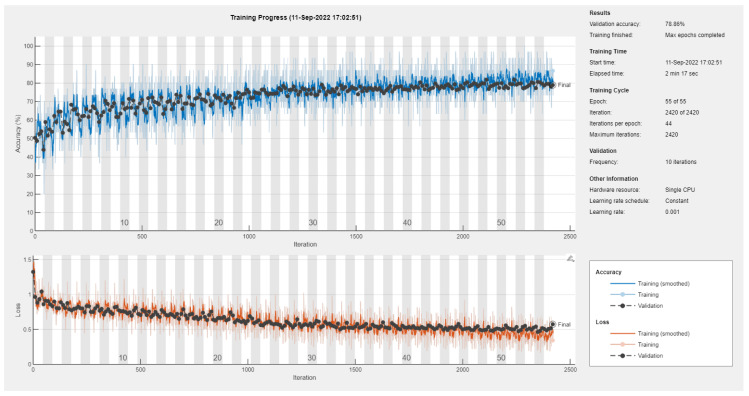
Training graph of the CNN model for the three-stress class based on the BH dataset.

**Figure 17:**
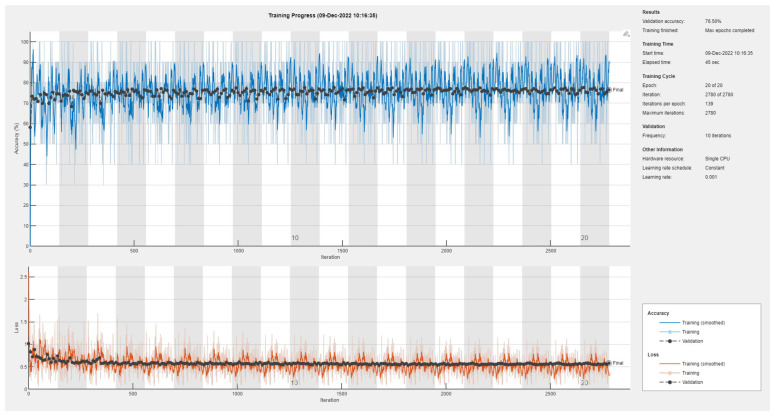
Training graph of the CNN model for the three-stress class based on the E4-L dataset.

**Figure 18:**
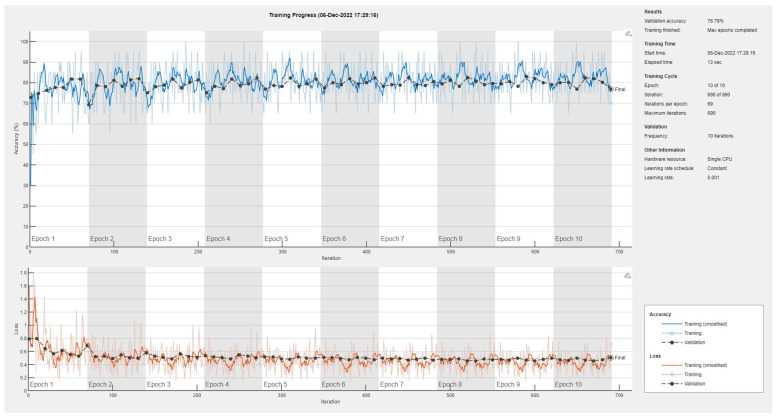
Training graph of the CNN model for the three-stress class based on the E4-R dataset.

**Figure 19:**
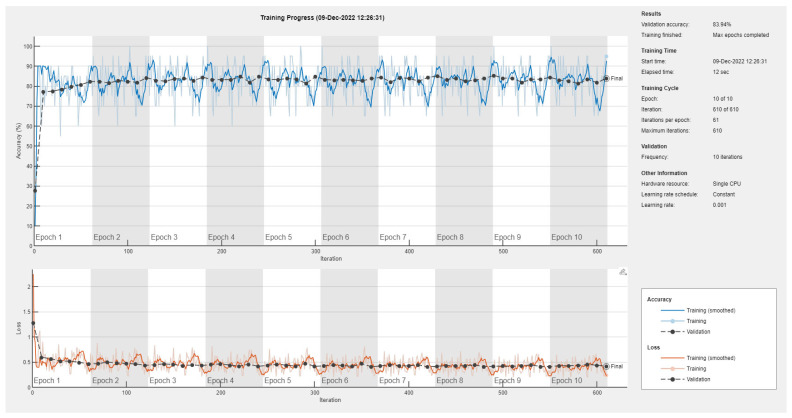
Training graph of the CNN model for the three-stress class based on the E4-(L+R) datasets.

**Figure 20:**
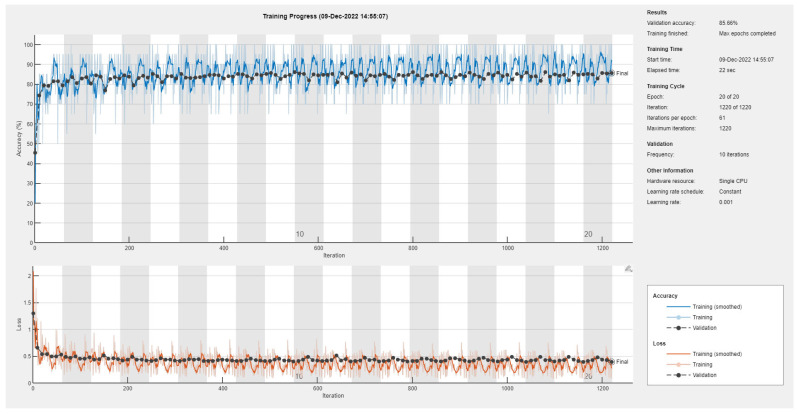
Training graph of the CNN model for the three-stress class based on the BH+E4-(L+R) datasets.

**Figure 21:**
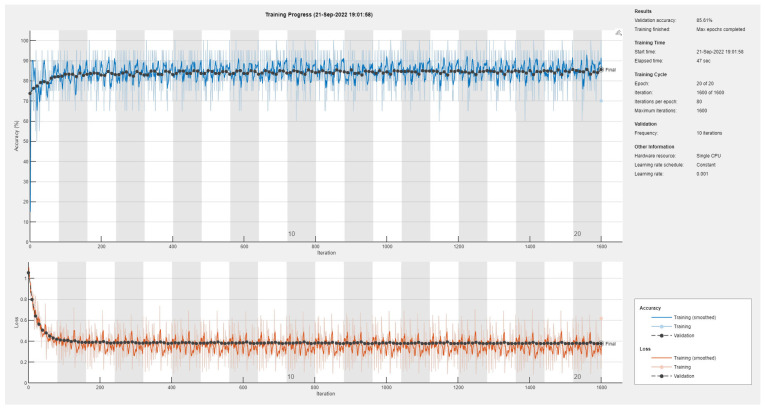
Training graph of the hybrid CNN-LSTM model for the three-stress class based on the SRAD dataset.

**Figure 22:**
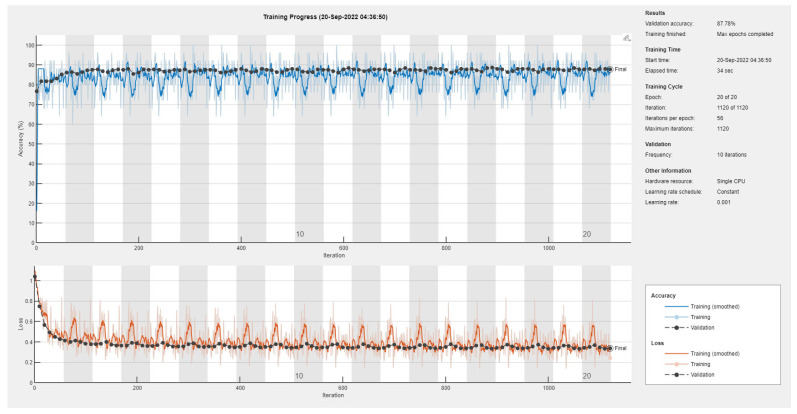
Training graph of the hybrid CNN-LSTM model for the three-stress class based on the BH dataset.

**Figure 23:**
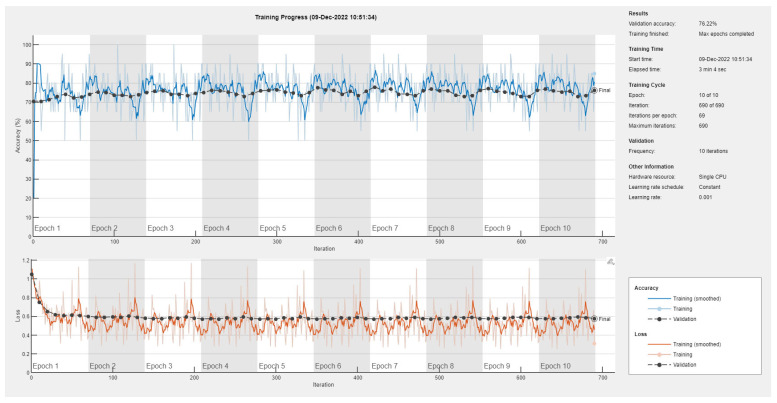
Training graph of the hybrid CNN-LSTM model for the three-stress class based on the E4-L dataset.

**Figure 24:**
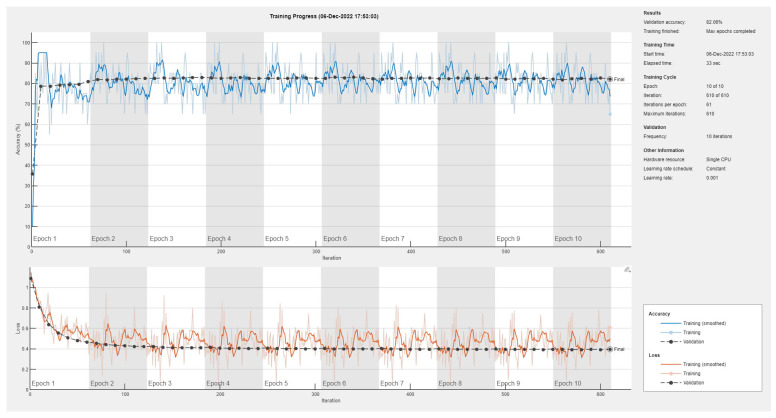
Training graph of the hybrid CNN-LSTM model for the three-stress class based on the E4-R dataset.

**Figure 25:**
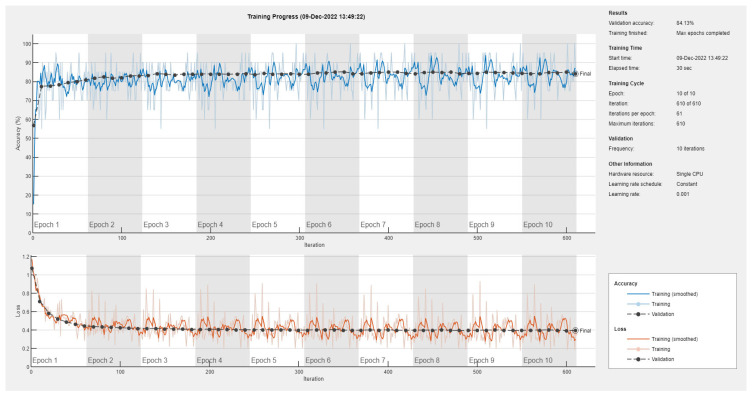
Training graph of the hybrid CNN-LSTM model for the three-stress class based on the E4-(L+R) datasets.

**Figure 26:**
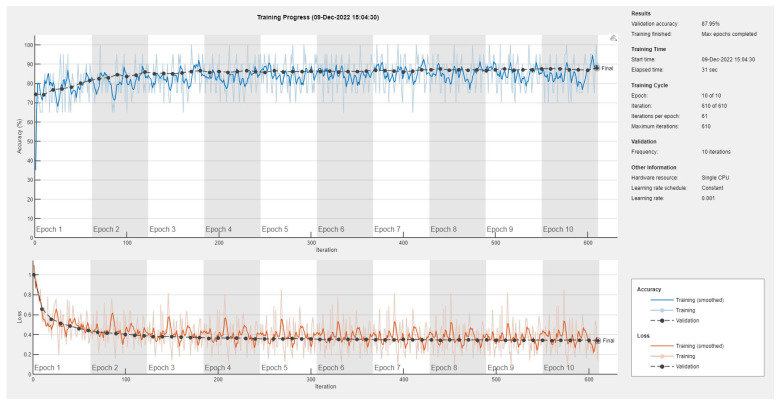
Training graph of the hybrid CNN-LSTM model for the three-stress class based on the BH+E4-(L+R) datasets.

Results of the proposed driver stress recognition models for the three-stress class are shown in Table 4. These results are based on the training data obtained for the SRAD and AffectiveROAD datasets for real-world driving. The training graphs of the proposed CNN models are shown in Figure 15, Figure 16, Figure 17, Figure 18, Figure 19 and Figure 20. Similarly, the training graphs of the proposed hybrid CNN-LSTM models are shown in Figure 21, Figure 22, Figure 23, Figure 24, Figure 25 and Figure 26. Results show that the proposed CNN model based on the BH+E4-(L+R) datasets outperform the other models based on the SRAD, BH, E4-L, E4-R, and E4-(L+R) datasets significantly by 6.16%, 6.76%, 9.16%, 8.87%, and 1.72%, respectively, with an overall validation accuracy of 85.66%. Similarly, the proposed hybrid CNN-LSTM model based on the BH+E4-(L+R) datasets outperform the other models based on the SRAD, BH, E4-L, E4-R, and E4-(L+R) datasets significantly by 2.15%, 0.15%, 11.22%, 5.89%, and 3.82%, respectively, with an overall validation accuracy of 87.95%. 

**Figure 27:**
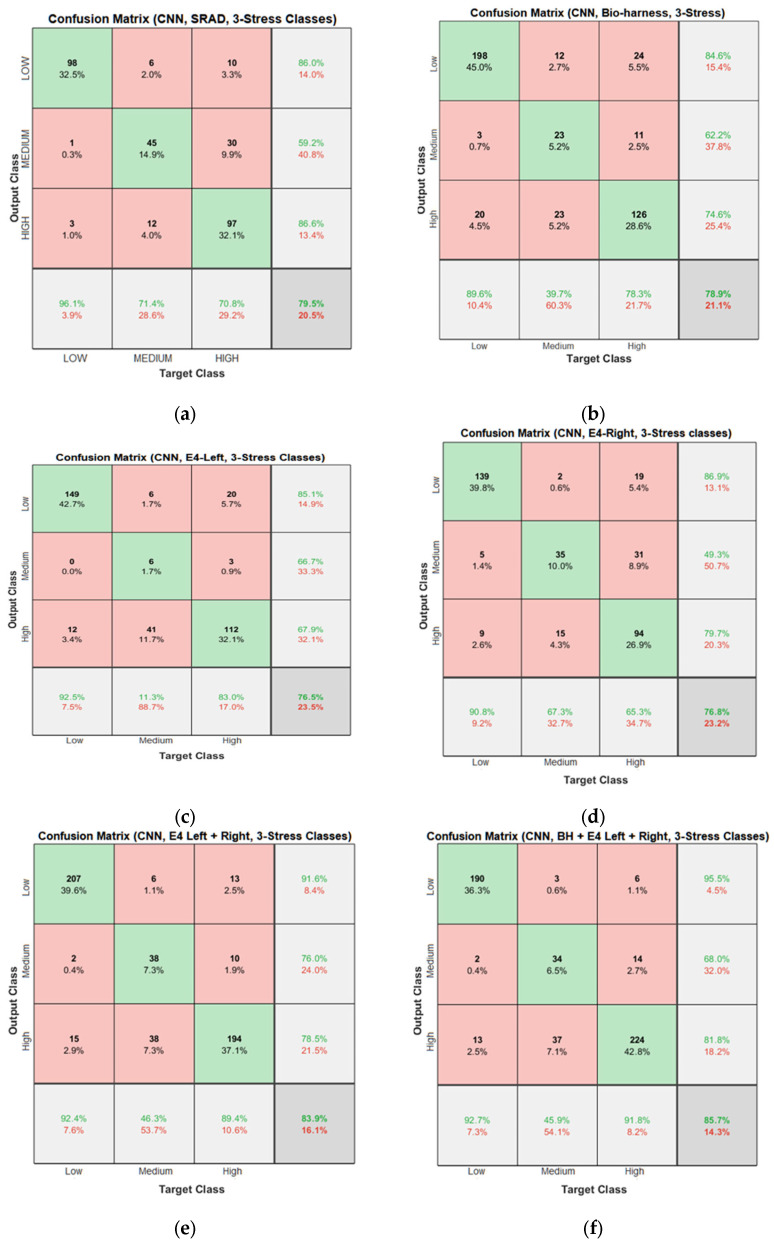
Confusion matrices of the CNN models for the three-stress class based on: (**a**) the SRAD dataset; (**b**) BH dataset; (**c**) E4-L dataset; (**d**) E4-R dataset; (**e**) E4-(L+R) datasets; (**f**) BH+E4-(L+R) datasets.

**Figure 28:**
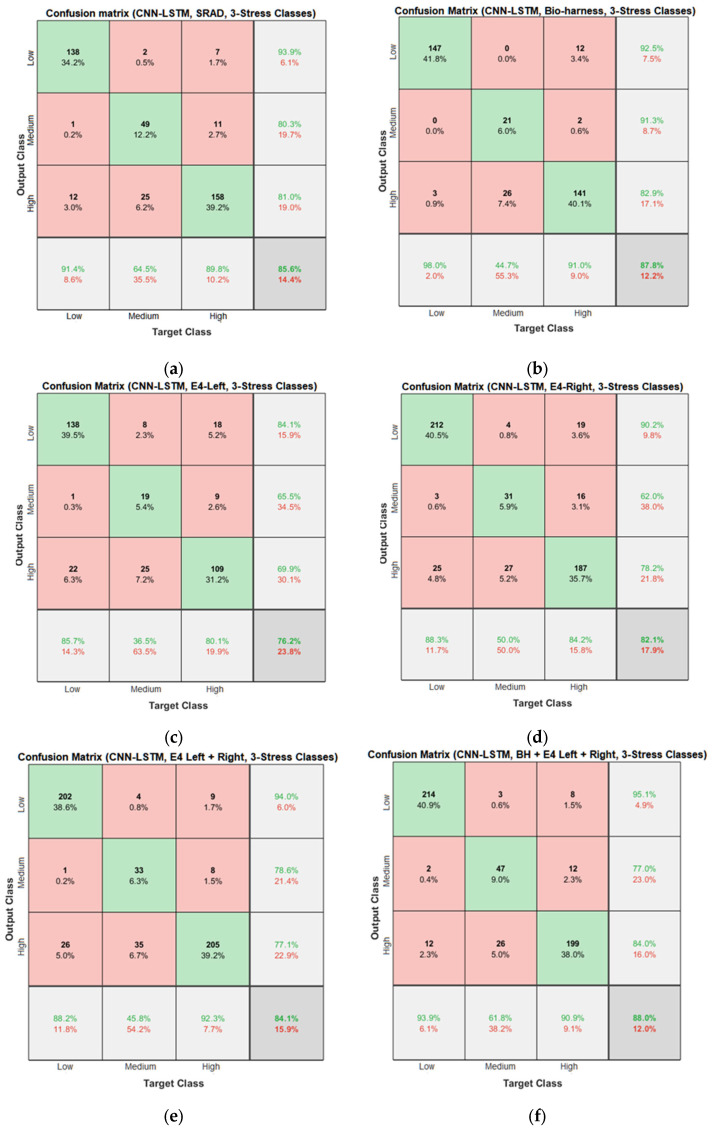
Confusion matrices of the hybrid CNN-LSTM models for the three-stress class based on: (**a**) the SRAD dataset; (**b**) BH dataset; (**c**) E4-L dataset; (**d**) E4-R dataset; (**e**) E4-(L+R) datasets; (**f**) BH+E4-(L+R) datasets.

Confusion matrices for the proposed CNN and hybrid CNN-LSTM models are shown in Figure 27 and Figure 28. In Figure 27f, 190 low instances are predicted correctly, while 3 and 6 low instances are incorrectly predicted as medium and high by the CNN model. So, the total correct prediction for the high-stress class is 95.5%. Likewise, for the medium- and high-stress classes, 34 out of 50 and 224 out of 274 instances were correctly predicted, which amounts to total accuracies of 68% and 81.8% for medium- and high-stress classes, respectively. Similarly, in Figure 28f, 214 low instances are predicted correctly, while 3 and 8 low instances are incorrectly predicted as medium and high by the CNN-LSTM model. Therefore, the total correct prediction for the low-stress class is 95.1%. Similarly, for the medium- and high-stress classes, 47 out of 61 and 199 out of 237 instances were correctly predicted, which amounts to total accuracies of 77% and 84% for the medium- and high-stress classes, respectively.

### 4.3. Rank-Based Performance Evaluation

The eight-step fuzzy EDAS procedure [65] defined in Section 3.6 is utilized here to evaluate the ranks of the SRAD, BH, E4-L, E4-R, E4-(L+R), and BH+E4-(L+R)-based CNN and hybrid CNN-LSTM models for the two-stress and three-stress classes. This procedure is separately followed for each the driver’s stress level. The classification metrics calculated in Table 3 and Table 4 are regarded as a criterion for the proposed CNN and hybrid CNN-LSTM driver stress level classification models for the two-stress and three-stress classes.

#### 4.3.1. Rank Estimation of the CNN Models for Two Levels of Stress

A rank estimation of the CNN models for the two-stress class (relaxed state) is performed in a series of steps. The results of each step are shown in Table 5, Table 6, Table 7, Table 8, Table 9 and Table 10. The first step determines the cross-efficient values $\left(\psi_{\beta }\right)$ using Equations (1) and (2), as shown in Table 5. In the next two steps, the positive distance $\left(P_{I}\right)$ and negative distance $\left(N_{I}\right)$ are separately determined based by Equations (5) and (8), as given in Table 6 and Table 7. In the fourth and fifth steps, the weighted sum of $\left(P_{I}\right)$ and $\left(N_{I}\right)$ are separately calculated with the help of Equations (9) and (10), as shown in Table 8 and Table 9. The sixth step normalizes the weighted sums ${{(SP}_{I})}_{\alpha }$ and ${{(SN}_{I})}_{\alpha }$ independently to obtain the aggregate scores of the models based on Equations (11) and (12), as indicated in Table 10. Finally, the appraisal score $\left(\lambda_{\alpha }\right)$ is determined based on the aggregate scores $N{{(SP}_{I})}_{\alpha }$ and $N{{(SN}_{I})}_{\alpha }$ in the seventh step with the help of Equation (13), as given in Table 10. The eighth step uses the appraisal scores $\left(\lambda_{\alpha }\right)$ to determine the ranks of the proposed CNN models based on the BH, E4-L, E4-R, E4-(L+R), and BH+E4-(L+R) datasets. The model with the lowest appraisal score $\left(\lambda_{\alpha }\right)$ has the highest performance among the candidate models. Table 10 shows that the proposed BH+E4-(L+R), E4-L, E4-R, SRAD, E4-(L+R), and BH-based CNN models achieved first, second, third, fourth, fifth, and fifth positions for the relaxed state. Likewise, the same eight-step procedure is utilized for the stressed state, and the resulting ranks of each CNN model are given in Table 11. Table 11 shows that the proposed BH+E4-(L+R), SRAD, E4-L, E4-R, E4-(L+R), and BH-based CNN models achieved first, second, third, fourth, fifth, and fifth positions for the stressed state.

#### 4.3.2. Rank Estimation of the CNN-LSTM Models for Two Levels of Stress

For the rank estimation of the SRAD, BH, E4-L, E4-R, E4-(L+R), and BH+E4-(L+R)-based hybrid CNN-LSTM models, the same eight-step procedure is utilized for the relaxed state and stressed state, and the resulting ranks are given in Table 12 and Table 13, respectively. Table 12 shows that the proposed BH+E4-(L+R), BH, E4-L, E4-R, E4-(L+R), and SRAD-based CNN-LSTM models achieved first, second, third, third, fourth, and fifth positions for the relaxed state. Similarly, Table 13 shows that the proposed fused BH+E4-(L+R), BH, E4-L, E4-R, SRAD, and E4-(L+R)-based CNN-LSTM models achieved first, second, third, third, fourth, and fifth positions for the stressed state.

#### 4.3.3. Rank Estimation of the CNN Models for Three Levels of Stress

The eight-step fuzzy EDAS procedure is also utilized for the three levels of stress (low, medium, and high) and the resulting ranks of the SRAD, BH, E4-L, E4-R, E4-(L+R), and BH+E4-(L+R)-based CNN models are shown in Table 14, Table 15 and Table 16, respectively. For the low-stress level, BH+E4-(L+R), SRAD, E4-(L+R), E4-L, E4-R, and BH-based CNN models achieved first, second, third, fourth, fifth, and fifth positions, as shown in Table 14. For the medium-stress level, E4-R, E4-(L+R), BH+E4-(L+R), E4-L, SRAD and BH-based CNN models achieved first, second, third, fourth, fifth, and fifth positions, as shown in Table 15. Likewise, Table 16 shows that the proposed BH+E4-(L+R), SRAD, E4-L, E4-R, BH, and E4-(L+R)-based CNN models achieved first, second, third, fourth, fifth, and fifth positions for the high-stress level.

#### 4.3.4. Rank Estimation of the CNN-LSTM Models for Three Levels of Stress

For the rank estimation of the SRAD, BH, E4-L, E4-R, E4-(L+R), and BH+E4-(L+R)-based hybrid CNN-LSTM models, the same eight-step procedure is utilized for low-stress, medium-stress, and high-stress, and the resulting ranks are given in Table 17, Table 18 and Table 19, respectively. Table 17 shows that the proposed BH+E4-(L+R), BH, E4-L, E4-R, SRAD, and E4-(L+R)-based CNN-LSTM models achieved first, second, third, third, fourth, and fifth positions for the low-stress level. Moreover, Table 18 shows that the proposed, SRAD, E4-R, E4-L, BH, and BH+E4-(L+R)-based CNN-LSTM models achieved first, second, third, third, fourth, and fifth positions for the medium-stress level. For the high-stress level, BH, BH+E4-(L+R), SRAD, E4-L, E4-R, and E4-(L+R)-based CNN models achieved first, second, third, third, fourth, and fifth positions, as shown in Table 19. 

### 4.4. Comparison of the Proposed 1D CNN and 1D CNN-LSTM Models

Comparisons of the proposed 1D CNN and 1D CNN-LSTM models for two and three levels of stress based on training time, accuracy, and fuzzy EDAS ranking are shown in Table 20, Table 21, Table 22 and Table 23. Execution environments for all proposed models are based on a single CPU. The fuzzy EDAS approach performs a comprehensive rank estimation of the proposed models in terms of accuracy, recall, precision, F-score, and specificity. The model with the lowest appraisal score $\left(\lambda_{\alpha }\right)$ has the highest performance among the candidate models. 

A comparison shows that there is a tradeoff between the training time and performance of various models, with the exception of the SRAD dataset. For example, Table 20 shows that the proposed BH+E4-(L+R)-based 1D CNN model achieved the best EDAS ranks at the cost of a maximum training time of 72 min and 42 s. On the other hand, the BH-based 1D CNN model has the worst EDAS rank with the lowest training time of 3 min and 15 s. The performance of the SRAD-based model is somehow in between with the highest computational cost of 277 min and 29 s. Similarly, Table 21 reveals that the proposed BH+E4-(L+R)-based 1D CNN-LSTM model secured the top EDAS ranks at the cost of a maximum training time of 72 min and 42 s. Moreover, the BH-based 1D CNN model has the worst EDAS rank with the lowest training time of 3 min and 15 s. However, the SRAD-based model achieved average performance, with a maximum computational cost of 167 min and 56 s.

Table 22 shows the comparison of the 1D CNN model for the three-stress class. As usual, the proposed BH+E4-(L+R) secured the best EDAS ranks at a maximum cost of 72 min and 51 s. On the other hand, the BH-based model has the worst EDAS rank for a minimum computational cost of 3 min and 45 s. However, the SRAD-based model has average performance with the highest training time of 279 min and 6 s. Similarly, Table 23 reveals that the proposed SRAD-based 1D CNN-LSTM model secured the top EDAS rank at the cost of a maximum training time of 171 min and 31 s. The BH-based 1D CNN-LSTM model achieved an average EDAS rank with the lowest training time of 2 min and 2 s. However, the E4-(L+R)-based model has the worst EDAS rank despite the high training time of 71 min and 9 s. The proposed models have a high training time due to the usage of a single CPU. Utilizing GPUs may reduce the training time of the proposed algorithms.

## 5. Discussion

The proposed CNN and hybrid CNN-LSTM models are analyzed using the SRAD and AffectiveROAD datasets in the previous section. AffectiveROAD is based on the BH, Empatica E4-L, Empatica E4-R, and E4-(L+R) datasets. Both BH and Empatica E4 datasets are individually and combinedly used to train the proposed models for the two-stress and three-stress classes. It is evident from the previous tables that the models trained on multimodal data (AffectiveROAD) achieved the maximum performance compared to SRAD data. This shows the importance of physiological, physical, and contextual information in the domain of stress recognition. Moreover, it is also clear that the hybrid 1D CNN-LSTM models achieved better performance than the 1D CNN models. The fuzzy EDAS procedure also shows that the proposed CNN and hybrid CNN-LSTM models achieved the first rank based on the fused AffectiveROAD BH+E4-(L+R) datasets. The achieved performance of the proposed real-world driver stress recognition models is greatly enhanced compared to the existing schemes. A comparison of the proposed driver stress recognition models with the existing schemes is shown in Table 24. It is clear from the table that the proposed models achieved the highest performance for both two and three levels of stress compared to the existing schemes. Rastgoo et al. [11] achieved a higher performance than the proposed models for the three-stress class but their study was based on simulated driving conditions, while the proposed models were based on real-world driving conditions.

## 6. Conclusions

This paper concludes that in addition to physiological signals, other information regarding the driver, vehicle, and ambiance has an important role in designing reliable and accurate driver stress recognition systems. It is also evident that the hybrid CNN-LSTM models have better performance than the CNN models. Moreover, the fusion of the AffectiveROAD datasets (BH, E4-L, and E4-R) achieved the best performance with the least computational cost compared to the SRAD dataset. This is due to several factors including the utilization of multimodal information (physiological signals and information regarding the driver, vehicle, and environment), quality of the hardware and software tools used for capturing the data, and accurate sampling of the signals. Thus, hybrid deep learning models and multimodal data have a key role in designing an accurate and reliable stress recognition model for real-world driving conditions.

The fusion models based on 1D CNN and hybrid 1D CNN-LSTM produced promising results, but these models may be further improved by utilizing more complex CNN and LSTM architectures. Moreover, a joint CNN-LSTM architecture may be used to further improve stress level recognition. The current study is based on the driver’s stress level, thus in the future, these models may be utilized for drowsiness, cognitive workload, activity, fatigue, and feeling recognition. The AffectiveROAD and PhysioNet SRAD datasets used in this study are based on real-world driving conditions. Such datasets are usually contaminated by different noises and artifacts. Enhanced pre-processing techniques can further improve the performance of the models. Future work may also include stress recognition by training the proposed models using physiological signals acquired using non-contact sensors and smart watches.

## Figures and Tables

**Table 1:** One-dimensional CNN architectures and parameter settings.

Dataset		Feature Learning (1D CNN)		Training Options (1D CNN)				
	Filter Size	Padding	Layers	Optimization Algorithm (Feature Learning/ Classification)	No. of Epochs (Feature Learning/ Classification)	Mini-Batch size (Feature Learning/ Classification)	Validation Frequency	Training/ Validation Set
SRAD	03	Causal	1st Bloch: Conv1D (Filters: 8), ReLU, LN 2nd: Bloch: Conv1D (Filters: 32), ReLU, LN 3rd Block: Conv1D (Filters: 64), ReLU, LN 4th Block: Conv1D (Filters: 128), ReLU, LN Conv1D (Filters: 8) GAP; FC; Softmax; Classification	Adam	20	30	10	80:20
E4-L					30 20	20 10		
E4-R					30 10	20 20		
E4-(L+R)			Sum of the layers of E4-L and E4-R		30 + 30 10	20 + 20 20		
BH			1st Bloch: Conv1D (Filters: 128), ReLU, LN 2nd: Bloch: Conv1D (Filters: 64), ReLU, LN 3rd Block: Conv1D (Filters: 32), ReLU, LN GAP; Softmax; FC; Classification		80 10	20 20		
BH+E4-(L+R)			Sum of the layers of BH, E4-L, and E4-R		80 + 30 + 30 10	20 + 20 + 20 20		

**Table 2:** One-dimensional CNN-LSTM architectures and parameter settings.

Dataset		Features Learning (1D CNN)		Training Options (1D CNN-LSTM)						Training/ Validation Set
	Filter Size	Padding	Layers	Hidden Layers	Dropout	Optimization Algorithm (Feature Learning/ Classification)	Epochs (Feature Learning/ Classification)	Mini-Batch Size (Feature Learning/ Classification)	Validation Frequency	
SRAD	03	Causal	1st Bloch: Conv1D (Filters: 8), ReLU, LN 2nd: Bloch: Conv1D (Filters: 32), ReLU, LN 3rd Block: Conv1D (Filters: 64), ReLU, LN 4th Block: Conv1D (Filters: 128), ReLU, LN Conv1D (8 Filters) GAP; FC; Softmax; Classification	250	0.4	Adam	20 20	30	10	80:20
E4-L				300	0.5		30 10	20 20		
E4-R				200	0.5		30 10	20 20		
E4-(L+R)			Sum of the layers of E4-L and E4-R	200	0.5		30 + 30 10	20 + 20 20		
BH			1st Bloch: Conv1D (Filters: 128), ReLU, LN 2nd: Bloch: Conv1D (Filters: 64), ReLU, LN 3rd Block: Conv1D (Filters: 32), ReLU, LN GAP; Softmax; FC; Classification	200	0.5		80 20	20 20		
BH+E4-(L+R)			Sum of the layers of BH, E4-L, and E4-R	200	0.5		80 + 30 + 30 15	20 + 20 + 20 20		

**Table 3:** Performance analysis of the proposed fusion models for the two-stress class.

Deep Learning Model	Dataset	Driver’s Stress Level	Performance Measure					
			ACC	RCL	PRC	F1	SPC	Overall ACC
1D CNN	SRAD	Relaxed	0.9271	0.8774	0.9118	0.8942	0.9541	92.7%
		Stressed	0.9271	0.9541	0.9350	0.9444	0.8774	
	BH	Relaxed	0.8909	0.9278	0.8267	0.8743	0.8654	89.1%
		Stressed	0.8909	0.8654	0.9454	0.9036	0.9278	
	E4-L	Relaxed	0.8653	0.8072	0.9394	0.8683	0.9363	86.53%
		Stressed	0.8653	0.9363	0.7989	0.8622	0.8073	
	E4-R	Relaxed	0.8825	0.8636	0.8693	0.8664	0.8974	88.3%
		Stressed	0.8825	0.8974	0.8929	0.8951	0.8636	
	E4-(L+R)	Relaxed	0.9235	0.9776	0.8617	0.916	0.8833	92.35%
		Stressed	0.9235	0.8833	0.9815	0.9298	0.9776	
	BH+E4-(L+R)	Relaxed	0.9564	0.9772	0.9224	0.949	0.9417	95.6%
		Stressed	0.9564	0.9417	0.9831	0.962	0.9772	
1D CNN-LSTM	SRAD	Relaxed	0.9180	0.9041	0.8742	0.8889	0.9261	91.8%
		Stressed	0.9181	0.9261	0.9444	0.9352	0.9041	
	BH	Relaxed	0.9545	0.9857	0.9079	0.9452	0.9340	95.5%
		Stressed	0.9545	0.9340	0.9900	0.9612	0.9857	
	E4-L	Relaxed	0.8882	0.9007	0.85	0.8746	0.8788	88.83%
		Stressed	0.8882	0.8788	0.9206	0.8992	0.9007	
	E4-R	Relaxed	0.9065	0.9099	0.8649	0.8868	0.9041	90.65%
		Stressed	0.9065	0.9041	0.9371	0.9203	0.9099	
	E4-(L+R)	Relaxed	0.9465	0.9555	0.9328	0.944	0.9384	94.65%
		Stressed	0.9465	0.9384	0.9593	0.9487	0.9555	
	BH+E4-(L+R)	Relaxed	0.9659	0.9395	0.9873	0.9628	0.9893	96.59%
		Stressed	0.9659	0.9893	0.9486	0.9685	0.9395	

**Table 4:** Performance analysis of the proposed fusion models for the three-stress class.

Deep Learning Model	Dataset	Driver’s Stress Level	Performance Measure					
			ACC	RCL	PRC	F1	SPC	Overall ACC
1D CNN	SRAD	Low	0.9338	0.8596	0.9607	0.9074	0.9787	79.5%
		Medium	0.8377	0.5921	0.7143	0.6475	0.9203	
		High	0.8377	0.8661	0.708	0.7791	0.7895	
	BH	High	0.8659	0.8461	0.8959	0.8703	0.8883	78.9%
		Medium	0.8886	0.6216	0.3965	0.4842	0.9131	
		Low	0.8227	0.7456	0.7826	0.7636	0.8708	
	E4-L	Low	0.8911	0.8514	0.9255	0.8869	0.931	76.5%
		Medium	0.8567	0.6667	0.1132	0.1935	0.8618	
		High	0.7822	0.6788	0.8296	0.7467	0.875	
	E4-R	Low	0.8997	0.8687	0.9085	0.8882	0.9259	76.79%
		Medium	0.8481	0.493	0.6731	0.5691	0.9388	
		High	0.788	0.7966	0.6528	0.7176	0.7835	
	E4-(L+R)	Low	0.9312	0.9159	0.9241	0.9200	0.9428	83.94%
		Medium	0.893	0.7600	0.4634	0.5758	0.907	
		High	0.8547	0.7854	0.894	0.8362	0.9167	
	BH+E4-(L+R)	Low	0.9541	0.9548	0.9268	0.9406	0.9537	85.66%
		Medium	0.8929	0.6800	0.4595	0.5484	0.9154	
		High	0.8662	0.8175	0.918	0.8649	0.9197	
1D CNN-LSTM	SRAD	Low	0.9454	0.9388	0.9139	0.9262	0.9492	85.6%
		Medium	0.9032	0.8033	0.6447	0.7153	0.9210	
		High	0.8635	0.8103	0.8977	0.8517	0.9135	
	BH	Low	0.9574	0.9245	0.9800	0.9515	0.9845	87.8%
		Medium	0.9204	0.9130	0.4468	0.6000	0.9210	
		High	0.8778	0.8294	0.9097	0.8677	0.9231	
	E4-L	Low	0.8596	0.8415	0.8571	0.8492	0.8757	76.22%
		Medium	0.8768	0.6552	0.3654	0.4691	0.8969	
		High	0.788	0.6987	0.8015	0.7466	0.8601	
	E4-R	Low	0.9027	0.9021	0.8833	0.8926	0.9031	82.06%
		Medium	0.9046	0.62	0.5	0.5536	0.9346	
		High	0.834	0.7824	0.8423	0.8113	0.8772	
	E4-(L+R)	Low	0.9235	0.9395	0.8821	0.9099	0.9123	84.13%
		Medium	0.9082	0.7857	0.4583	0.5789	0.9189	
		High	0.8509	0.7707	0.9234	0.8402	0.9338	
	BH+E4-(L+R)	Low	0.9522	0.9511	0.9386	0.9448	0.953	87.95%
		Medium	0.9178	0.7705	0.6184	0.6861	0.9372	
		High	0.8891	0.8397	0.9087	0.8728	0.9301	

**Table 5:** Cross-efficient values of the CNN models for the relaxed state.

Dataset	Performance Measure (Driver’s Relaxed State)				
	ACC	RCL	PRC	F1	SPC
SRAD	0.9271	0.8774	0.9118	0.8942	0.9541
BH	0.8909	0.9278	0.8267	0.8743	0.8654
E4-L	0.8653	0.8072	0.9394	0.8683	0.9363
E4-R	0.8825	0.8636	0.8693	0.8664	0.8974
E4-(L+R)	0.9235	0.9776	0.8617	0.916	0.8833
BH+E4-(L+R)	0.9564	0.9772	0.9224	0.949	0.9417
$\psi_{\beta }$	0.9076	0.9051	0.8886	0.8947	0.9130

**Table 6:** Analysis results of the average ${(P}_{I})$ of the CNN models for the relaxed state.

Dataset	Performance Measure (Driver’s Relaxed State)				
	ACC	RCL	PRC	F1	SPC
SRAD	0.0000	0.0306	0.0000	0.0006	0.0000
BH	0.0184	0.0000	0.0696	0.0228	0.0522
E4-L	0.0466	0.1082	0.0000	0.0295	0.0000
E4-R	0.0277	0.0459	0.0217	0.0249	0.0171
E4-(L+R)	0.0000	0.0000	0.0302	0.0000	0.0326
BH+E4-(L+R)	0.0000	0.0000	0.0000	0.0000	0.0000

**Table 7:** Analysis results of the average ${(N}_{I}$ ) of the CNN models for the relaxed state.

Dataset	Performance Measure (Driver’s Relaxed State)				
	ACC	RCL	PRC	F1	SPC
SRAD	0.0000	0.0000	0.0262	0.0000	0.0450
BH	0.0000	0.0250	0.0000	0.0000	0.0000
E4-L	0.0000	0.0000	0.0572	0.0000	0.0255
E4-R	0.0000	0.0000	0.0000	0.0000	0.0000
E4-(L+R)	0.0175	0.0801	0.0000	0.0238	0.0000
BH+E4-(L+R)	0.0537	0.0796	0.0191	0.0607	0.0314

**Table 8:** Analysis results of the aggregate ${(P}_{I})$ of the CNN models for the relaxed state.

**Weight of Criteria**	0.4176	0.2850	0.14533	0.0844	0.0676	
**Dataset**	**Performance Measure (Driver’s Relaxed State)**					
	ACC	RCL	PRC	F1	SPC	${{(SP}_{I})}_{\alpha }$
SRAD	0.0000	0.0087	0.0000	0.0005	0.0000	0.0088
BH	0.0077	0.0000	0.0100	0.0019	0.0035	0.0233
E4-L	0.0000	0.0000	0.0000	0.0000	0.0000	0.0000
E4-R	0.0116	0.0000	0.0031	0.0027	0.0012	0.0185
E4-(L+R)	0.0000	0.0000	0.0044	0.0000	0.0022	0.0066
BH+E4-(L+R)	0.0000	0.0000	0.0000	0.0000	0.0000	0.0000

**Table 9:** Analysis results of the aggregate ${(N}_{I})$ of the CNN models for the relaxed state.

**Weight of Criteria**	0.4176	0.2850	0.1453	0.0844	0.0676	
**Dataset**	**Performance Measure (Driver’s Relaxed State)**					
	ACC	RCL	PRC	F1	SPC	${{(SN}_{I})}_{\alpha }$
SRAD	0.0090	0.0000	0.0038	0.0000	0.0030	0.0158
BH	0.0000	0.0071	0.0000	0.0000	0.0000	0.0071
E4-L	0.0000	0.0000	0.0083	0.0000	0.0017	0.0100
E4-R	0.0000	0.0000	0.0000	0.0000	0.0000	0.0000
E4-(L+R)	0.0073	0.0228	0.0000	0.0020	0.0000	0.0321
BH+E4-(L+R)	0.0224	0.0227	0.0055	0.0051	0.0021	0.0579

**Table 10:** Analysis results of the CNN models for the relaxed state.

Dataset	${{(SP}_{I})}_{\alpha }$	${{(SN}_{I})}_{\alpha }$	$N{{(SP}_{I})}_{\alpha }$	$N{{(SN}_{I})}_{\alpha }$	$\lambda_{\alpha }$	Ranks
SRAD	0.0088	0.0158	0.3774	0.7270	0.5522	4
BH	0.0233	0.0071	1.0000	0.8768	0.9384	6
E4-L	0.0000	0.0100	0.0000	0.8267	0.4133	2
E4-R	0.0000	0.0000	0.0000	1.0000	0.5000	3
E4-(L+R)	0.0185	0.0321	0.7968	0.4452	0.6210	5
BH+E4-(L+R)	0.0066	0.0579	0.2835	0.0000	0.1417	1

**Table 11:** Analysis results of the CNN models for the stressed state.

Dataset	${{(SP}_{I})}_{\alpha }$	${{(SN}_{I})}_{\alpha }$	$N{{(SP}_{I})}_{\alpha }$	$N{{(SN}_{I})}_{\alpha }$	$\lambda_{\alpha }$	Ranks
SRAD	0.0021	0.0263	0.0874	0.4791	0.2832	2
BH	0.0237	0.0052	1.0000	0.8960	0.9480	6
E4-L	0.0000	0.0073	0.0000	0.8562	0.4280	3
E4-R	0.0000	0.0000	0.0000	1.0000	0.5000	4
E4-(L+R)	0.0213	0.0232	0.8985	0.5401	0.7193	5
BH+E4-(L+R)	0.0093	0.0505	0.3913	0.0000	0.1956	1

**Table 12:** Analysis results of the CNN-LSTM models for the relaxed state.

Dataset	${{(SP}_{I})}_{\alpha }$	${{(SN}_{I})}_{\alpha }$	$N{{(SP}_{I})}_{\alpha }$	$N{{(SN}_{I})}_{\alpha }$	$\lambda_{\alpha }$	Ranks
SRAD	0.0214	0.0000	1.0000	1.0000	1.0000	5
BH	0.0000	0.0311	0.0000	0.2328	0.1164	2
E4-L	0.0000	0.0000	0.0000	1.0000	0.5000	3
E4-R	0.0000	0.0000	0.0000	1.0000	0.5000	3
E4-(L+R)	0.0212	0.0225	0.9887	0.4452	0.7169	4
BH+E4-(L+R)	0.0000	0.0405	0.0000	0.0000	0.0000	1

**Table 13:** Analysis results of the CNN-LSTM models for the stressed state.

Dataset	${{(SP}_{I})}_{\alpha }$	${{(SN}_{I})}_{\alpha }$	$N{{(SP}_{I})}_{\alpha }$	$N{{(SN}_{I})}_{\alpha }$	$\lambda_{\alpha }$	Ranks
SRAD	0.0093	0.0000	0.5875	1.0000	0.7938	4
BH	0.0000	0.0247	0.0000	0.3496	0.1748	2
E4-L	0.0000	0.0000	0.0000	1.0000	0.5000	3
E4-R	0.0000	0.0000	0.0000	1.0000	0.5000	3
E4-(L+R)	0.0158	0.0145	1.0000	0.6195	0.8097	5
BH+E4-(L+R)	0.0000	0.0380	0.0000	0.0000	0.0000	1

**Table 14:** Analysis results of the CNN models for the low-stress level.

Dataset	${{(SP}_{I})}_{\alpha }$	${{(SN}_{I})}_{\alpha }$	$N{{(SP}_{I})}_{\alpha }$	$N{{(SN}_{I})}_{\alpha }$	$\lambda_{\alpha }$	Ranks
SRAD	0.0075	0.0190	0.1696	0.5996	0.3846	2
BH	0.0441	0.0000	1.0000	1.0000	1.0000	6
E4-L	0.0000	0.0003	0.0000	0.9937	0.4968	4
E4-R	0.0000	0.0000	0.0000	1.0000	0.5000	5
E4-(L+R)	0.0104	0.0214	0.2357	0.5504	0.3931	3
BH+E4-(L+R)	0.0000	0.0476	0.0000	0.0000	0.0000	1

**Table 15:** Analysis results of the CNN models for the medium-stress level.

Dataset	${{(SP}_{I})}_{\alpha }$	${{(SN}_{I})}_{\alpha }$	$N{{(SP}_{I})}_{\alpha }$	$N{{(SN}_{I})}_{\alpha }$	$\lambda_{\alpha }$	Ranks
SRAD	0.0348	0.1006	1.0000	0.0000	0.5000	5
BH	0.0322	0.0094	0.9250	0.9061	0.9155	6
E4-L	0.0000	0.0140	0.0000	0.8240	0.4120	4
E4-R	0.0000	0.0761	0.0000	0.2438	0.1219	1
E4-(L+R)	0.0103	0.0793	0.2956	0.2117	0.2537	2
BH+E4-(L+R)	0.0022	0.0392	0.0638	0.6101	0.3370	3

**Table 16:** Analysis results of the CNN models for the high-stress level.

Dataset	${{(SP}_{I})}_{\alpha }$	${{(SN}_{I})}_{\alpha }$	$N{{(SP}_{I})}_{\alpha }$	$N{{(SN}_{I})}_{\alpha }$	$\lambda_{\alpha }$	Ranks
SRAD	0.0224	0.0371	0.3835	0.4636	0.4236	2
BH	0.0194	0.0009	0.3326	0.9868	0.6597	5
E4-L	0.0000	0.0071	0.0000	0.8974	0.4487	3
E4-R	0.0000	0.0054	0.0000	0.9212	0.4606	4
E4-(L+R)	0.0584	0.0439	1.0000	0.3648	0.6824	6
BH+E4-(L+R)	0.0000	0.0691	0.0000	0.0000	0.0000	1

**Table 17:** Analysis results of the CNN-LSTM models for the low-stress level.

Dataset	${{(SP}_{I})}_{\alpha }$	${{(SN}_{I})}_{\alpha }$	$N{{(SP}_{I})}_{\alpha }$	$N{{(SN}_{I})}_{\alpha }$	$\lambda_{\alpha }$	Ranks
SRAD	0.0000	0.0204	0.0000	0.4467	0.2233	4
BH	0.0000	0.0368	0.0000	0.0000	0.0000	2
E4-L	0.0000	0.0000	0.0000	1.0000	0.5000	3
E4-R	0.0000	0.0000	0.0000	1.0000	0.5000	3
E4-(L+R)	0.0173	0.0072	1.0000	0.8034	0.9017	5
BH+E4-(L+R)	0.0058	0.0332	0.3365	0.0981	0.2173	1

**Table 18:** Analysis results of the CNN-LSTM models for the medium-stress level.

Dataset	${{(SP}_{I})}_{\alpha }$	${{(SN}_{I})}_{\alpha }$	$N{{(SP}_{I})}_{\alpha }$	$N{{(SN}_{I})}_{\alpha }$	$\lambda_{\alpha }$	Ranks
SRAD	0.0009	0.0732	0.0559	0.0000	0.0280	1
BH	0.0170	0.0653	1.0000	0.1072	0.5536	4
E4-L	0.0000	0.0000	0.0000	1.0000	0.5000	3
E4-R	0.0000	0.0009	0.0000	0.9870	0.4935	2
E4-(L+R)	0.0085	0.0118	0.4974	0.8383	0.6678	6
BH+E4-(L+R)	0.0168	0.0561	0.9891	0.2327	0.6109	5

**Table 19:** Analysis results of the CNN-LSTM models for the high-stress level.

Dataset	${{(SP}_{I})}_{\alpha }$	${{(SN}_{I})}_{\alpha }$	$N{{(SP}_{I})}_{\alpha }$	$N{{(SN}_{I})}_{\alpha }$	$\lambda_{\alpha }$	Ranks
SRAD	0.0000	0.0196	0.0000	0.5913	0.2956	3
BH	0.0000	0.0379	0.0000	0.2113	0.1057	1
E4-L	0.0000	0.0000	0.0000	1.0000	0.5000	4
E4-R	0.0000	0.0000	0.0000	1.0000	0.5000	4
E4-(L+R)	0.0187	0.0102	1.0000	0.7884	0.8942	5
BH+E4-(L+R)	0.0064	0.0480	0.3450	0.0000	0.1725	2

**Table 20:** Comparison of the proposed CNN models for the two-stress class.

Dataset	Execution Environment	Training Time 1D CNN		Performance				
				Accuracy (%)			Fuzzy EDAS Rank	
		Feature Learning	Classification	Relaxed State	Stressed State	Overall	Relaxed State	Stressed State
SRAD	Single CPU	277 min 29 s		92.71	92.71	92.72	4	2
E4-L		35 min, 18 s	13 s	86.53	86.53	86.53	2	3
E4-R		35 min, 26 s	13 s	88.25	88.25	88.25	3	4
E4-(L+R)		70 min, 26 s	12 s	92.35	92.35	92.35	5	5
BH		1 min, 46 s	1 min, 29 s	89.09	89.09	89.09	6	6
BH+E4-(L+R)		72 min, 30 s	12 s	95.64	9564	95.64	1	1

**Table 21:** Comparison of the proposed CNN-LSTM models for the two-stress class.

Dataset	Execution Environment	Training Time 1D CNN-LSTM		Performance				
				Accuracy (%)			Fuzzy EDAS Rank	
		Feature Learning	Classification	Relaxed State	Stressed State	Overall	Relaxed State	Stressed State
SRAD	Single CPU	167 min 33 s	23 s	91.80	91.80	91.81	5	4
E4-L		35 min 18 s	34 s	88.82	88.82	88.83	3	3
E4-R		35 min 26 s	31 s	90.65	90.65	90.65	3	3
E4-(L+R)		70 min 26 s	32 s	94.65	94.65	94.65	4	5
BH		1 min 46 s	33 s	95.45	95.45	95.45	2	2
BH+E4-(L+R)		72 min 30 s	21 s	96.59	96.59	96.59	1	1

**Table 22:** Comparison of the proposed CNN models for the three-stress class.

Dataset	Execution Environment	Training Time 1D CNN		Performance						
				Accuracy (%)				Fuzzy EDAS Rank		
		Feature Learning	Classification	Low-stress State	Medium-Stress State	High-Stress State	Overall	Low-Stress State	Medium-Stress State	High-Stress State
SRAD	Single CPU	279 min, 6 s		93.38	83.77	83.77	79.47	2	5	2
E4-L		35 min, 20 s	45 s	89.11	85.67	78.22	76.50	4	4	3
E4-R		35 min, 19 s	13 s	89.97	84.81	78.80	76.79	5	1	4
E4-(L+R)		70 min, 39 s	12 s	93.12	89.30	85.47	83.94	3	2	6
BH		1 min, 28 s	2 min, 17 s	86.59	88.86	82.27	78.86	6	6	5
BH+E4-(L+R)		72 min, 7 s	22 s	95.41	89.29	86.62	85.66	1	3	1

**Table 23:** Comparison of the proposed CNN-LSTM models for the three-stress class.

Dataset	Execution Environment	Training Time 1D CNN-LSTM		Performance						
				Accuracy (%)				Fuzzy EDAS Rank		
		Feature Learning	Classification	Low-Stress State	Medium-Stress State	High-Stress State	Overall	Low-Stress State	Medium-Stress State	High-Stress State
SRAD	Single CPU	170 min, 44 s	47 s	94.54	90.32	86.35	85.61	4	1	1
E4-L		35 min, 20 s	3 min, 4 s	85.96	87.68	78.80	76.22	3	3	3
E4-R		35 min, 19 s	33 s	90.27	90.46	83.40	82.06	3	2	2
E4-(L+R)		70 min, 39 s	30 s	92.35	90.82	85.09	84.13	5	6	6
BH		1 min, 28 s	34 s	95.74	92.04	87.78	87.78	2	4	4
BH+E4-(L+R)		72 min, 7 s	31 s	95.22	91.78	88.91	87.95	1	5	5

**Table 24:** Comparison of the proposed stress recognition models with existing schemes.

Article / Year	Signal(s) / Modalities	Environment	No. of Subjects	Data I/P Mechanism	Deep Learning Approach	Stress Levels	ACC (%)	RCL	PRC	F1	SPC
Proposed Models	HR, BR, Posture, Activity, TEMP, IBI, GSR, ACCL	Real-World Driving	10	1D Signal	CNN-LSTM	2	96.6	96.4	96.8	96.6	96.4
						3	88	85.4	82.2	83.5	94
[50]/2021	HR, GSR		9	Continues RPs	CNN	2	95.7	95.7	95.9	95.7	-
[46]/2019	Facial Images		123	Images	Pre-Trained MTCNN	2	97.3	-	-	-	-
[11]/2019	ECG, VDD, EP	Simulated Driving	27	1D Signal	CNN-LSTM	3	92.8	94.1	95	-	97.4
[49]/2016	EEG		37	1D Signal	CCNN	2	86.1	-	-	-	-
[53]/2019	RESP and ECG	Cognitive Tasks (Lab/Workplace)	18	1D Signal	CNN-LSTM	3	83.9	-	-	81.1	-
[56]/2021	EEG		32	Spectrogram	Pre-Trained AlexNet	2	84.8	85.2	-	-	84.3
[55]/2019	ECG		20		CNN	2	82.7	-	-	-	-
[54]/2018	ECG		13	1D Signal	1D CNN	2	80	-	-	-	-
[52]/2017	Thermographic Patterns of Breath		8	Spectrogram	CNN	2	84.6	-	-	-	-

## Data Availability

The data will be available on request.
